# Dynamic Transcriptomic and Metabolomic Analyses of *Madhuca pasquieri* (Dubard) H. J. Lam During the Post-germination Stages

**DOI:** 10.3389/fpls.2021.731203

**Published:** 2021-09-30

**Authors:** Lei Kan, Qicong Liao, Zhipeng Chen, Shuyu Wang, Yifei Ma, Zhiyao Su, Lu Zhang

**Affiliations:** College of Forestry and Landscape Architecture, South China Agricultural University, Guangzhou, China

**Keywords:** *Madhuca pasquieri* (Dubard) H. J. Lam, post-germination stages, transcriptomics, widely-targeted metabolomics, flavonols biosynthesis, polar auxin transport

## Abstract

The wild population of *Madhuca pasquieri* (Dubard) H. J. Lam is currently dwindling; its understory seedlings are rare, and there is a lack of molecular studies, which impedes the conservation of this species. This study exploited second-generation sequencing and widely targeted metabolomics analysis to uncover the dynamic changes in differentially expressed genes (DEGs) and differentially accumulated metabolites (DAMs) in five post-germination stages of *M. pasquieri* whole organism. Notably, the weighted gene co-expression network analysis (WGCNA), transcriptome, and metabolome association analyses all indicated significant enrichment of the flavonoid biosynthesis pathway in stage 4 (two-leaf), and an upregulation of the genes encoding flavonol biosynthesis in this stage. In stage 5 (nine-leaf), the flavonols were significantly accumulated, indicating that the changes in metabolites were driven at the transcript level. According to the significant changes in gene expression encoding auxin transport carriers and their correlation with flavonols during stage 5, the flavonols were speculated to have a direct inhibitory effect on the expression of PIN4 encoding gene, which may inhibit the process of polar auxin transport. The results provided important insights into the molecular network relationships between the transcription and metabolism of this rare and endangered species during the post-germination stages and explained the reasons for the slow growth of its seedlings at the molecular level.

## Introduction

Seed germination is the start of the plant life cycle (Bewley, 1997), and the development after germination has a straightforward influence on plant survival (Li et al., 2005). Under the influence of environment, during post-germination, the growth is complicated by various morphological (Romero-Rodriguez et al., 2018), physiological (Qu et al., 2019a), and biochemical changes (Wang et al., 2020). Extensive studies have been conducted on both physiological and morphological levels of post-germination in herbaceous plants, such as maize (*Zea mays*) (Anzala et al., 2006), soybean (*Glycine max* L.) (Gronwald et al., 2009), rice (*Oryza sativa* L.) (Ho et al., 2013), and wheat (*Triticum aestivum* L.) (Sun et al., 2020).

According to the International Union for the Conservation of Nature (IUCN) Red List, *Madhuca pasquieri* (Dubard) H. J. Lam is regarded as a vulnerable (VU) species in the Sapotaceae family. In China, it has been recorded as a national key protected wild plant (II) of tiny population. These trees mainly grow in mixed forests or mountain forest edges below the height of 1,100 m in southern China and northern Vietnam (Flora of China (FOC), 2021). *Madhuca pasquieri* is not only a rare woody oil tree but also a precious timber species. The current research on *M. pasquieri* mainly focuses on *in-situ, ex-situ* protection, chemical composition, and artificial cultivation, and is still in the primary stage. Based on the previous investigation of the authors on the population of this species, we found that its native habitat was seriously fragmented; its seedlings in the understory were very rare, and were difficult to regenerate. We also found that the growth of *M. pasquieri* was very slow during the artificial cultivation in the post-germination stages. Although in the previous study, PacBio combined with an Illumina platform was used to obtain reference sequence through full-length transcriptome sequencing of *M. pasquieri* (Kan et al., 2020), there is still a lack of research on the growth of *M. pasquieri* in post-germination stages at the molecular level.

“Omics” methods have been used in recent years to obtain knowledge of the alterations of metabolites, proteins, and gene transcripts (Wedow et al., 2019). Gene expression can be detected with transcriptome methods, whereas functional changes caused by these genes or proteins can be investigated by metabolomics (Yuan et al., 2018), which is an effective way to analyze the complicated process of post-germination growth. Multi-omics analysis has been a powerful method to identify correlations between genes and metabolites (Saito, 2013). Using transcriptome and integrated metabolome to study the biological process of poplar (*Poplar simonii* × *Poplar nigra*) post-germination growth, it was found that cell wall, amino acid metabolism, and transport-related pathways were obviously enriched during cotyledon expansion, while primary metabolic processes were not (Qu et al., 2019b). Combined transcriptome and metabolome analyses were performed on mung bean (*Vigna radiata*) and seedlings at three time points: 6 h, 3 days, and 6 days (seed germination, hypocotyl elongation, and epicotyl elongation). A lot of transcript changes occurred between samples from seed germination and hypocotyl elongation, including starch and sucrose metabolism, glycolysis, plant hormone regulation, and amino acid synthesis. Additionally, the alterations in metabolites were also detected, including carbohydrates and amino acids, indicating it was driven by the altered genes expressions (Wang et al., 2020). In another poplar study, it was found that during the growth process from the early seed germination stage to the post-germination stage, genes related to CHO metabolism were activated first, followed by gene expression related to lipid metabolism, and then protein metabolism, and changes in metabolites further verified the sequence of these biological events (Qu et al., 2019a). Widely targeted metabolomics is a new method that can accurately detect hundreds of target metabolites, and is broadly used in plants, e.g., *Arabidopsis* (*Arabidopsis thaliana*) (Sawada et al., 2017), rice (Yang et al., 2019b), and apple (*Malus domestica*) (Xu et al., 2020). Moreover, it has proven that the combination analysis of metabolome and transcriptome data can effectively reveal the biosynthetic mechanisms of the main metabolic pathway of post-germination growth in plants (Yang et al., 2020). Therefore, the combination of widely targeted metabolomics and transcriptomics is very necessary for the in-depth understanding of the post-germination growth of *M. pasquieri*. The integration of modern omics techniques provides a comprehensive perspective to better understand the biological processes of post-germination events in plants at the molecular level.

Investigating the molecular mechanism of slow growth in the post-germination of *M. pasquieri* could clearly elucidate the reasons why this species is endangered. Seed germination, by definition, begins when mature dry seeds absorb water, and ends when the radicle protrudes through the seed envelope (Bewley, 1997). With the development of the tree growth, we divided and defined it as five stages, namely stages 1–15: the seed germination stage, which is the last stage of germination, subsequent hypocotyl elongation stage, epicotyl elongation stage, two-leaf stage, and nine-leaf stage. Using Illumina RNA-seq and ultra-performance liquid chromatography-tandem mass spectrometry (UPLC-MS/MS) technologies, we obtained transcriptome and metabolome data from the five post-germination stages in the whole organism of *M. pasquieri*. Weighted gene co-expression network analysis (WGCNA) was performed to identify stage-specific gene clusters, network modules, and module key genes of differentially expressed genes (DEGs) between each stage. Then, combined with the metabolome data, an association analysis was performed to a construct transcript-metabolite correlation network, and the flavonol synthesis pathway and polar auxin transport process were further analyzed. From the perspective of transcription and metabolism, this study explored the reasons for the slow growth of *M. pasquieri* post-germination, which provided new insight for the in-depth analysis of post-germination growth and functions of *M. pasquieri*, and a molecular basis for the protection of this species in the future.

## Materials and Methods

### Plant Materials

**Figure 1:**
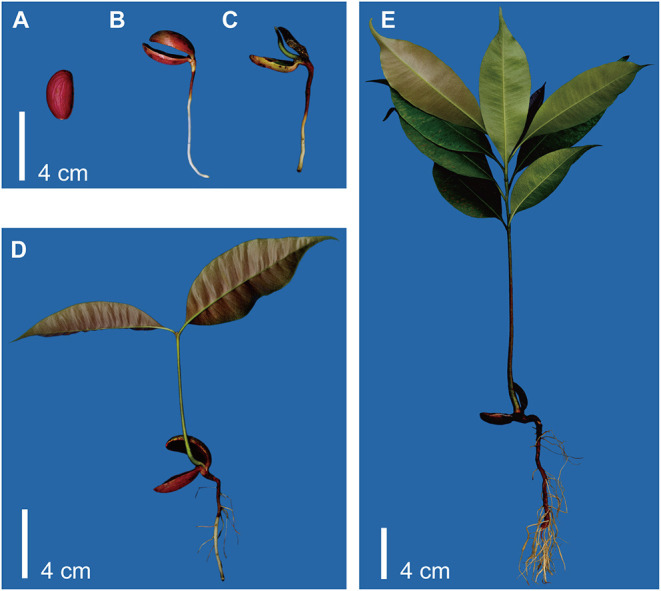
Schematic representation of the experimental setup. **(A)** Stage 1: seed germination. **(B)** Stage 2: hypocotyl elongation. **(C)** Stage 3: epicotyl elongation. **(D)** Stage 4: two-leaf stage. **(E)** Stage 5: nine-leaf stage.

*Madhuca pasquieri* was grown in an artificial climate chamber (RXZ-500C-LED; Ningbo Jiangnan Instrument Factory, Zhejiang, China), at a temperature of 25°C, humidity of 60–80%, and a light cycle of 14/10 h (day/night), 17,600 lx, at the South China Agricultural University. *Madhuca pasquieri* plants were selected based on the five developmental stages from the same batch of light matrix culture in the artificial climate chamber (seed germination, hypocotyl elongation, epicotyl elongation, two-leaf, and nine-leaf stages; Figure 1) during post-germination growth, with three biological replicates per stage. The collected whole organism samples were snap-frozen in liquid nitrogen and stored at −80°C until use.

### Widely Targeted Metabolome Detection and Data Analysis

The freeze-dried whole organism samples from the five developmental stages, with three biological replicates per stage, were crushed using a mixer mill (MM 400; RETSCH, Haan, Germany) with a zirconia bead for 1.5 min at 30 Hz. Then, 100 mg powder of homogenized tissue was extracted overnight at 4°C with 1 ml 70% aqueous methanol (Merck, Darmstadt, Germany; www.merckchemicals.com) containing 0.1 mg/l lidocaine for the internal standard. Following centrifugation at 10,000 g for 10 min, the supernatant was absorbed and filtrated (SCAA-104, 0.22 μm pore size; ANPEL, Shanghai, China; www.anpel.com.cn/) before liquid chromatography-tandem mass spectrometry (LC-MS/MS) analysis. Quality control (QC) samples were mixed by all the samples to detect the reproducibility of the whole experiment. The repeatability of metabolite extraction and detection was judged by the overlapping analysis of the total ion current (TIC) in the different QC samples (Supplementary Figure 1). The TIC curves overlapped during metabolite detection, and the retention times and peak intensities were consistent, indicating that the signals of the identical sample were stable at a different detection time.

The compounds extracted were analyzed using an LC-ESI-MS/MS system [UPLC, Shim-pack UFLC Shimadzu CBM30A system; Shimadzu, Kyoto, Japan; http://www.shimadzu.com.cn/; MS/MS (6500 Q TRAP; Applied Biosystems, Waltham, MA, United States; http://www.appliedbiosystems.com.cn/) (Chen et al., 2013). Data filtering, peak detection, alignment, and calculations were performed using the Analyst 1.6.1 (AB SCIEX, USA) software. The metabolites were identified by searching the internal and public databases MassBank (Horai et al., 2010), KNApSAcK (Nakamura et al., 2013), HMDB (Wishart et al., 2013), MoTo DB (Grennan, 2009), and METLIN (Zhu et al., 2013)), and comparing the m/z values, RT, and fragmentation patterns with the standards. The standards were divided into two levels. The standard of Level A was that the m/Z and RT were consistent with the database substances, and the matching score of the secondary mass spectrometry was more than 90. Level B meant that the matching score of the secondary mass spectrometry was between 60 and 90 when the above parameters were checked with the database. Both levels were for known substances and were scored using MasterView Software.

Metabolite abundances were quantified using the peak areas. To preliminarily visualize the differences between different groups, an unsupervised dimensionality reduction method principal component analysis (PCA) was performed in all the samples using R package models (http://www.r-project.org/). Partial least squares discriminant analysis (PLS-DA) is a supervised dimensionality reduction method in which class memberships are coded in the matrix form into Y to better distinguish the metabolomics profile of two groups by screening variables correlated to class memberships. Orthogonal least partial squares discriminant analysis (OPLS-DA) is derived from PLS-DA. Compared with PLS-DA, OPLS-DA is a combination of orthogonal signal correction (OSC) and PLS-DA (Westerhuis et al., 2008). The data obtained from the metabolite profiling were normalized for the PCA and OPLS-DA. The differentially accumulated metabolites (DAMs) were identified using a combination of variable importance in the projection (VIP) score of the OPLS model and Student's *t*-test. Those with a *P*-value of *t*-test < 0.05 and VIP ≥ 1 were considered as differential metabolites between the two groups.

### Transcriptome Profiling and Analysis

Whole organisms of *M. pasquieri* plants sampled at five developmental stages, with three biological replicates per stage, were used for the Illumina RNA sequencing. After the total RNA was extracted, the eukaryotic mRNA with a poly-A tail was enriched with Oligo (dT) beads, and then the enriched mRNA was fragmented into short fragments by ultrasonic waves and reverse-transcribed into cDNA using random primers. The second-strand cDNA was synthesized with DNA polymerase I, RNase H, dNTP, and a buffer (New England Biolabs, Ipswich, MA, United States). Next, the cDNA fragments were purified using a QiaQuick PCR extraction kit (Qiagen, Düsseldorf, Germany) and end-repaired, the poly-A was added, and the fragments were then ligated to the Illumina sequencing adapters. The ligation products were size-selected by agarose gel electrophoresis, amplified by PCR, and sequenced using Illumina HiSeq™ 4000 by Gene Denovo Biotechnology Company (Guangzhou, China).

Reads obtained from the sequencing machines included raw reads containing adapters or low-quality bases, which affect subsequent assembly and analysis. Thus, fastp (version 0.18.0) was applied to obtain high-quality clean reads by further filtering according to the following rules (Chen et al., 2018): (1) removal of reads containing adapters; (2) removal of reads containing more than 10% of unknown nucleotides (N); (3) removal of reads containing all A bases; (4) removal of low-quality reads containing more than 50% low-quality (Q-value ≤ 20) bases. The high-quality clean reads were mapped to the ribosomal RNA (rRNA) to identify the residual rRNA reads. The rRNA-removed reads were used for further analysis.

The rRNA-removed high-quality clean reads were mapped to the reference transcriptome of *M. pasquieri* (SRP267710, https://www.ncbi.nlm.nih.gov/sra/15293472) using a short reads alignment tool, Bowtie2 (Johns Hopkins University, Baltimore, Maryland, United States) (Li et al., 2009) by default parameters, and mapping ratio was calculated.


$$\begin{array}{l} \;Mapping\;ratio\;=(Unique\;mapped\;reads\;number\\ +\;Multiple\;mapped\;reads\;numbers)/All\;read\;number \end{array}$$

Principal component analysis was also performed with R package models (http://www.r-project.org/) in this study. To identify DEGs across the groups, the edgeR package (http://www.r-project.org/) was used. The DEGs were identified with a fold change ≥2 and a false discovery rate (FDR) <0.05 by comparison. The fragments per kilobase of transcript per million mapped (FPKM) reads of each gene were calculated and used to quantify the expression level of the annotated genes.

### Gene Ontology (GO) Enrichment Analysis

Gene Ontology enrichment analysis provides all GO terms that are significantly enriched in DEGs compared with the genome background and filters of the DEGs that correspond to biological functions. First, the GOseq R package was applied to perform GO enrichment analysis, and all the DEGs were mapped to GO terms in the Gene Ontology database (http://www.geneontology.org/), gene numbers were calculated for every term, significantly enriched GO terms in DEGs compared with the genome background were defined by Wallenius' non-central hypergeometric distributions (Young et al., 2010). GO categories with FDR *q* ≤ 0.05 were considered to be significantly enriched.

### Kyoto Encyclopedia of Genes and Genomes (KEGG) Pathway Analysis

The Kyoto Encyclopedia of Genes and Genomes (KEGG, https://www.kegg.jp/kegg/) is the major public pathway-related database that links genomic or transcriptomic contents of genes to chemical structures of endogenous molecules (Kanehisa et al., 2008), thus providing a method to perform integration analysis of genes and metabolites. All the differentially expressed genes and metabolites in the study were mapped to the KEGG pathway database, and KOBAS 2.0 with hypergeometric tests was used to perform the KEGG enrichment analysis (Xie et al., 2011). The significance of KEGG pathway enrichment was determined with FDR *q* ≤ 0.05. Pathways meeting this condition were defined as significantly enriched in DEGs or DAMs.

### Weighted Gene Co-expression Network Analysis (WGCNA)

Co-expression networks were constructed using the WGCNA (v1.47) package in R (Langfelder and Horvath, 2008). Genes that were not expressed in more than half of the samples were filtered. After the filtration of low-expression genes, the gene expression values were imported into WGCNA to construct co-expression modules using the automatic network construction function blockwiseModules with default settings, except that the power is 7, mergeCutHeight power is 0.2, and minModuleSize power is 50. Genes were clustered into 15 correlated modules. Module eigengene (ME) values were calculated for each module and used to test for association with each stage. Networks were visualized using Cytoscape v.3.7.1 (Shannon et al., 2003).

### Transcriptome and Metabolome Correlation Network

Pearson's correlation coefficients were calculated based on the gene expression level (FPKM) of the transcriptome and the relative content of metabolites to obtain the correlation between metabolome and transcriptome data. Gene and metabolite pairs were ranked in the descending order of absolute correlation coefficients.

### Real-Time Quantitative Polymerase Chain Reaction (RT-qPCR)

The same RNA samples used in RNA-Seq were used in a real-time quantitative polymerase chain reaction (RT-qPCR). According to the instructions of the reverse transcription kit (R223; Vazyme Biotech, Nanjing, China), a 20-μl reaction system was established with 50 ng−2 μg total RNA and was incubated at 50°C for 50 min and 95°C for 5 min to obtain cDNAs. The cDNAs were then loaded in a 96-well plate for qRT-PCR analysis using StepOnePlus (ABI, CA, United States) with an RT-PCR reagent (Q341; Vazyme Biotech, Nanjing, China). The 20-μl reaction system consisted of 10 μl of 2 × ChamQ SYBR qPCR Master Mix (Vazyme Biotech, Nanjing, China), 0.4 μl of a PCR forward primer (10 μM), 0.4 μl of a PCR reverse primer (10 μM), 4 μl of a cDNA template, and 5.2 μl of ddH_2_O. PCR conditions were as follows: 95°C for 90 s, 40 cycles of 95°C for 5 s, 60°C for 15 s, and 72°C for 20 s. Relative gene expression levels were analyzed according to the 2^−ΔΔCt^ method. Product specificity and reaction efficiencies were verified for each primer pair. The primer pairs are listed in Supplementary Table 1.

## Results

### Metabolite Analysis

In the definition of the traditional seed germination stage, the termination of germination is the protuberance of the radicle through the seed envelope, so in this study, we define this stage as stage 1 (seed germination). According to the post-germination stages, hypocotyl elongation, epicotyl elongation, two-leaf stage, and nine-leaf stage were defined as stages 2 to 5, respectively. First, we prepared the whole organism samples at five stages of *M. pasquieri*, as shown in Figure 1.

**Figure 2:**
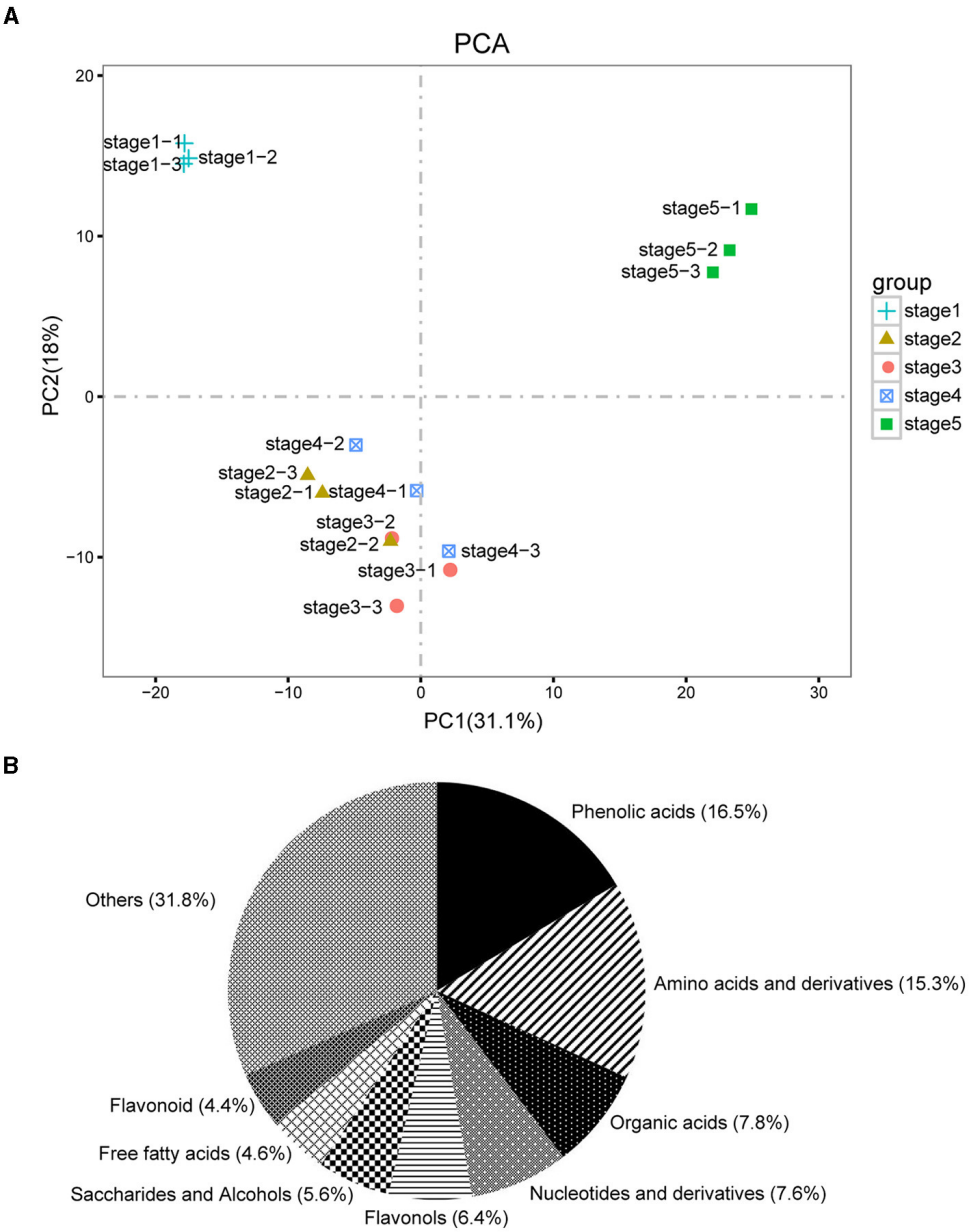
Overall qualitative and quantitative analyses of the metabolomics data. **(A)** Principal component analysis (PCA) of the five development stage samples and quality control samples (mixed); the x-axis represents the first principal component, and the y-axis represents the second principal component. **(B)** Component analysis of the identified metabolites. The top eight metabolites are shown beside the graph.

The dynamic metabolite changes in the five different stages of *M. pasquieri* were evaluated by UPLC-MS/MS. By qualification control and repeatability analysis, we showed the stability of the instruments and ensured the reliability and repeatability of the metabonomic data. PCA was performed to show the overall metabolic differences between inner and inter-group variations. As shown in Figure 2A, the samples from stage 1 and stage 5 gathered into a distinct cluster, respectively, while the samples from stages 2, 3, and 4 had an obvious overlap. However, OPLS-DA showed that there was still a clear separation between any two comparison stages (Supplementary Figure 2). These results indicated that the data were reproducible enough to be used in subsequent analyses.

In the available data, there were 497 metabolites that had been identified in the five development stages of *M. pasquieri* with three biological replicates in each stage (Supplementary Table 2). Of the 497 metabolites, phenolic acids (16.5%), amino acids and derivatives (15.3%), organic acids (7.8%), nucleotides and derivatives (7.6%), flavonols (6.4%), and saccharides and alcohols (5.6%) accounted for a large proportion (Figure 2B). DAMs were identified according to the standard of VIP ≥ 1 of OPLS-DA and *P* < 0.05 of *t*-test between each pairwise comparison. In total, 51, 57, 65, and 80 DAMs were identified (Table 1). For stage 1 vs. stage 2, 24 DAMs were upregulated and 27 were downregulated, and most of the upregulated DAMs were lipids, tannins, and flavonoids; the downregulated DAMs were mainly amino acids and derivatives. For stage 1 vs. stage 3, 30 DAMs were upregulated and 27 were downregulated; the upregulated DAMs were mainly tannins, flavonoids, and terpenoids, and the downregulated DAMs were mainly amino acids and derivatives. Of the 65 DAMs in stage 1 vs. stage 4, 38 and 27 DAMs were upregulated and downregulated, respectively. Among them, the upregulated DAMs were mainly flavonoids and phenolic acids, and the downregulated DAMs were mainly amino acids and derivatives. Of the 80 DAMs in stage 1 vs. stage 5, 36, and 44 DAMs were upregulated and downregulated, respectively. Among them, most of the upregulated DAMs were flavonoids, phenolic acids, and lipids; the downregulated DAMs were also mainly amino acids and derivatives (Supplementary Table 3). The DAM accumulation patterns in the different groups were also evaluated by hierarchical cluster analysis (Supplementary Figure 3).

**Table 1:** Summary of differentially accumulated metabolites (DAMs) between stage 1 and the other groups (stages 2–5).

**Group Name**	**Number Up-regulated**	**Number Down-regulated**	**Number of differential metabolites**
Stage 1 vs. stage 2	24	27	51
Stage 1 vs. stage 3	30	27	57
Stage 1 vs. stage 4	38	27	65
Stage 1 vs. stage 5	36	44	80

*Stage 1, seed germination; stage 2, hypocotyl elongation; stage 3, epicotyl elongation; stage 4, two-leaf stage; stage 5, nine-leaf stage*.

**Figure 3:**
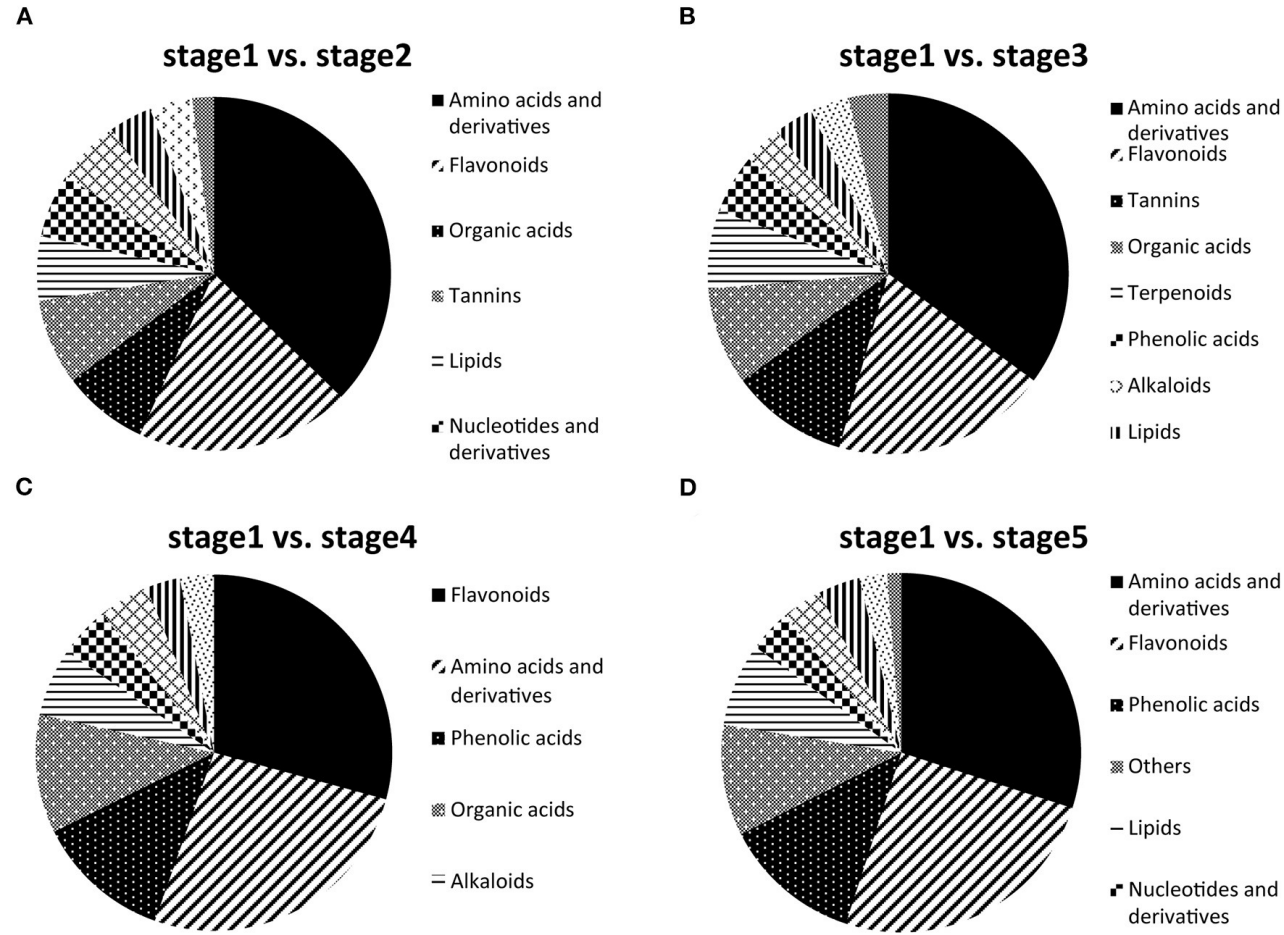
Differentially accumulated metabolites (DAMs) between stage 1 and the other stages (2–5). **(A)** Component analysis of DAMs in stage 1 vs. stage 2. **(B)** Component analysis of DAMs in stage 1 vs. stage 3. **(C)** Component analysis of DAMs in stage 1 vs. stage 4. **(D)** Component analysis of DAMs in stage 1 vs. stage 5.

Of these DAMs, amino acids and derivatives (37.25%), flavonoids (19.61%), organic acids (7.84%), tannins (7.84%), lipids (5.88%), nucleotides and derivatives (5.88%), and phenolic acids (5.88%) had a relatively large proportion in stage 2 compared with stage 1 (Figure 3A). Amino acids and derivatives (35.09%), flavonoids (19.30%), tannins (10.53%), organic acids (8.77%), and terpenoids (7.02%) were differentially accumulated in stages 1 and 3 (Figure 3B). Of the DAMs in stage 1 vs. stage 5, amino acids and derivatives (30%), flavonoids (25%), phenolic acids (12.5%), and other metabolites (10%) had the highest representation (Figure 3D). Interestingly, flavonoids (29.23%), amino acids and derivatives (26.15%), phenolic acids (12.31%), organic acids (10.77%), and alkaloids (6.15%) accounted for a large proportion in stages 1 and 4 (Figure 3C). To explore the functions of post-germination-related metabolites, the DAMs in stage 1 vs. stage 2, stage 1 vs. stage 3, stage 1 vs. stage 4, and stage 1 vs. stage 5 were functionally annotated using the KEGG database. For stage 1 vs. stage 2, the terms “aminoacyl-tRNA biosynthesis,” “glucosinolate biosynthesis,” and “biosynthesis of amino acids” were dominantly enriched (Supplementary Figure 4A). The terms “aminoacyl-tRNA biosynthesis,” “valine, leucine, and isoleucine degradation” and “alanine, aspartate, and glutamate metabolism” were enriched in stage 1 vs. stage 3 (Supplementary Figure 4B). However, only the term “valine, leucine, and isoleucine degradation” was significantly enriched in stage 1 vs. stage 4 (Supplementary Figure 4C). Meanwhile, for stage 1 vs. stage 5, the DAMs were strongly related to the terms “aminoacyl-tRNA biosynthesis,” “cyanoamino acid metabolism,” and “ABC transporters” (Supplementary Figure 4D).

### Global Analysis of RNA-seq Data and WGCNA

**Figure 4:**
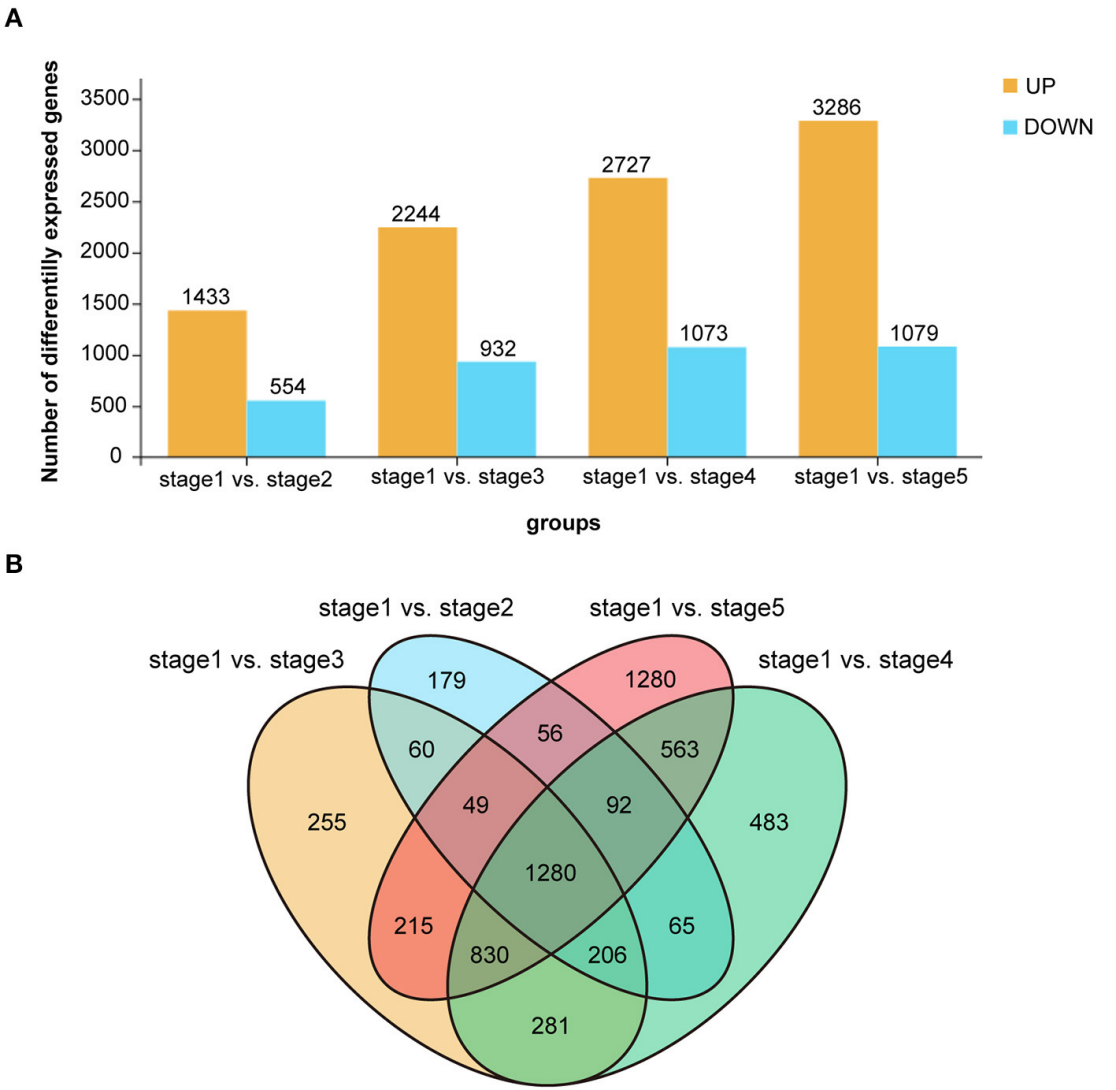
Differentially expressed genes (DEGs) of *Madhuca pasquieri* (Dubard) H. J. Lam during the post-germination stages. **(A)** Summary of the number of significantly changed transcripts between successive stages during *M. pasquieri* post-germination stages. **(B)** Venn diagram showing the overlap of DEGs during *M. pasquieri* post-germination stages.

Subsequently, we performed RNA-Seq to detect transcriptome differences among the post-germination stages of *M. pasquieri*. For each stage of the samples, an average of 45.6 million raw reads per library was detected (Table 2). All the reads in this study were mapped to the *M. pasquieri* reference transcriptome after mapping to the ribosome (Kan et al., 2020). In 15 libraries, approximately 54.23–68.65% of all the reads could be mapped on the reference transcriptome. Finally, a total of 22,685 genes, which correspond to 89.53% of the 25,339 genes predicted in the *M. pasquieri* transcriptome (Kan et al., 2020), were found to be expressed during the post-germination stages (Table 2). The PCA showed that the contribution rate of the first two primary components was 73.1%, and that the clustering of the samples in stage 1 and stage 5 was obvious, while the samples in the other three stages were relatively scattered and partially overlapped (Supplementary Figure 5). However, the overall grouping trend was consistent with the PCA result of the metabolome samples. In this study, we set FDR < 0.05 and |log2FC| > 1 (FC: fold change) as cut-off for screening DEGs. Here, 1,987 DEGs (1,433 up, 554 down) between stages 1 and 2 were detected; 3,176 DEGs (2,244 up, 932 down) between stages 1 and 3 were detected; 3,800 DEGs (2,727 up, 1,073 down) between stages 1 and 4 were detected; 4,365 DEGs (3,286 up, 1,079 down) between stages 1 and 5 were detected. The number of genes with significantly changed expression during *M. pasquieri* post-germination increased gradually, as shown in Figure 4A. A total of 5,894 DEGs were detected in the post-germination stages (stages 2–5) compared with the seed germination stage (stage 1), 1,280 DEGs were identified differentially expressed between stage 1 and the other development stages; 179, 255, 483, and 1,280 DEGs were specifically expressed in stages 2–5, respectively (Figure 4B). To classify the genes involved in the different development stages, KEGG pathway and GO enrichment analyses were performed for DEGs in stage 1 vs. stage 2, stage 1 vs. stage 3, stage 1 vs. stage 4, and stage 1 vs. stage 5. The terms “metabolic pathways” (ko01100), “biosynthesis of secondary metabolites” (ko01110), and “photosynthesis—antenna proteins” (ko00196) were all significantly enriched in each of the four other stages compared with stage 1 (Supplementary Figure 6). The GO analysis indicated that the DEGs of the different stages in biological process were mainly enriched for terms “metabolic process” (GO:0008152), “cellular process” (GO:0009987), and “single-organism process” (GO:0044699). For cellular component, the DEGs were mainly involved in “cell” (GO:0005623) and “cell part” (GO:0044464) in each stage compared with stage 1. The DEGs involved in molecular function mainly composed of “catalytic activity” (GO:0003824), “binding” (GO:0005488), and “transporter activity” (GO:0005215) in the different stages (Supplementary Table 4).

**Table 2:** Statistics of ribonucleic acid sequencing (RNA-Seq) reads obtained from the 15 samples and mapping to the *Madhuca pasquieri* (Dubard) H. J. Lam reference transcriptome.

**Sample**	**Low quality(%)**	**All reads number (%)**	**Mapped reads (%)**	**Genes number (%)**
Stage 1-1	181244 (0.20%)	42154302 (93.50%)	26796006 (63.57%)	17575 (69.36%)
Stage 1-2	155184 (0.19%)	38114744 (95.12%)	23373476 (61.32%)	17498 (69.06%)
Stage 1-3	162132 (0.21%)	36015022 (91.84%)	22075196 (61.29%)	17432 (68.80%)
Stage 2-1	231632 (0.23%)	48029318 (95.71%)	32195188 (67.03%)	18274 (72.12%)
Stage 2-2	220308 (0.27%)	38762952 (93.73%)	24676470 (63.66%)	18563 (73.26%)
Stage 2-3	201792 (0.19%)	49637142 (94.76%)	34075820 (68.65%)	18376 (72.52%)
Stage 3-1	152348 (0.19%)	39303962 (97.36%)	21313352 (54.23%)	18289 (72.18%)
Stage 3-2	257772 (0.29%)	42831964 (96.82%)	28085726 (65.57%)	18286 (72.17%)
Stage 3-3	179708 (0.21%)	41001822 (97.70%)	26498838 (64.63%)	18066 (71.30%)
Stage 4-1	191608 (0.22%)	41645196 (96.09%)	25515568 (61.27%)	18112 (71.48%)
Stage 4-2	181812 (0.18%)	47917986 (95.97%)	31312242 (65.35%)	18339 (72.37%)
Stage 4-3	279144 (0.32%)	41537518 (95.34%)	25755626 (62.01%)	18354 (72.43%)
Stage 5-1	185552 (0.20%)	42246108 (93.45%)	26543010 (62.83%)	18781 (74.12%)
Stage 5-2	214664 (0.20%)	52505450 (96.79%)	33765504 (64.31%)	19303 (76.18%)
Stage 5-3	193572 (0.19%)	49037022 (95.64%)	31349528 (63.93%)	19252 (75.98%)

*All read numbers: Unmapped read numbers of rRNA*.

*Mapped reads: Mapped read numbers of reference genes (ratio = mapped read/all read number)*.

*Gene number: Number of genes expressed in each sample (ratio = gene number/total reference gene number)*.

**Figure 5:**
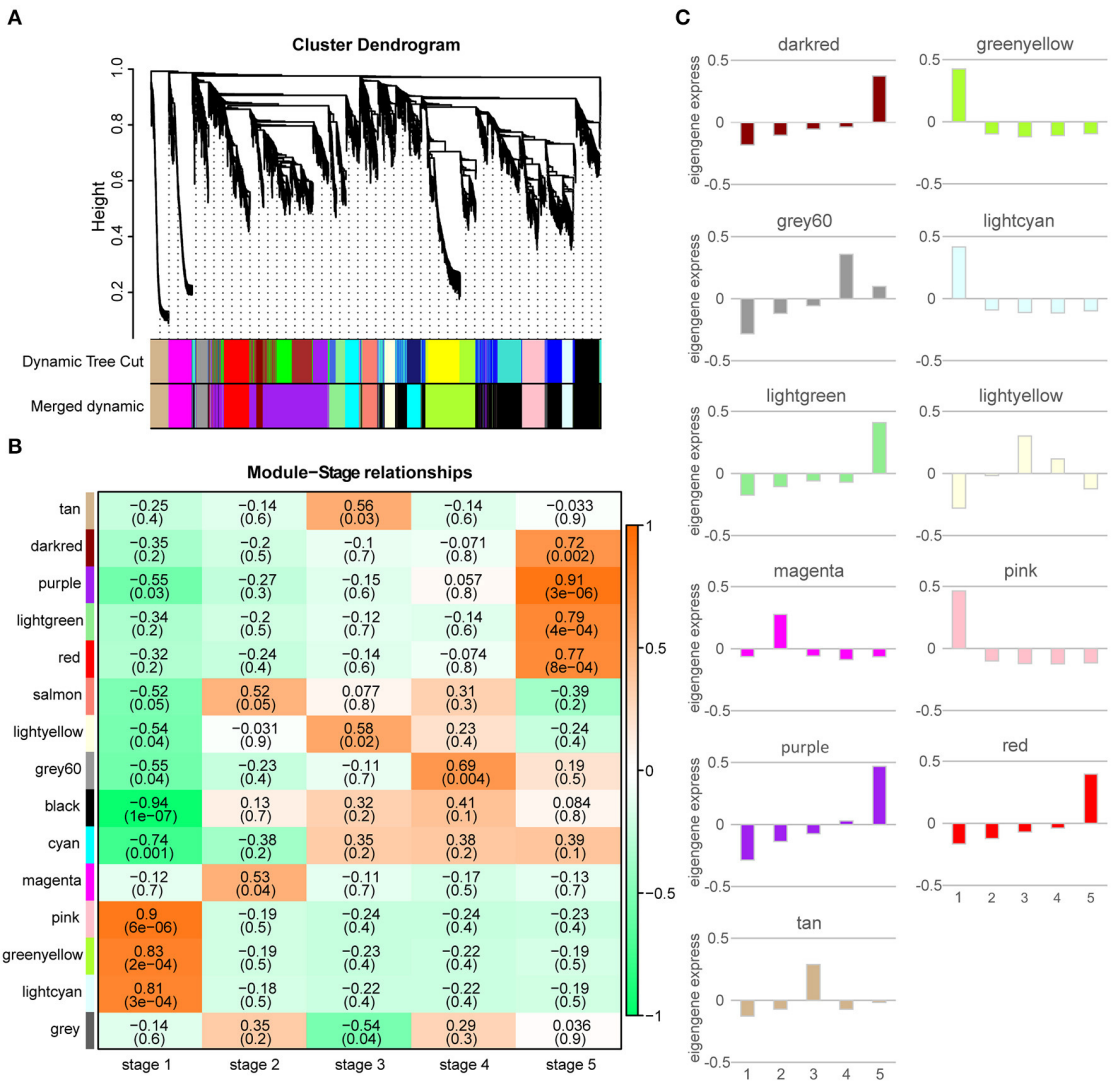
Weighted gene co-expression network analysis (WGCNA) of the significantly changed transcripts. **(A)** Hierarchical cluster tree showing co-expression modules and merged modules identified by the WGCNA. Each leaf in the tree is one gene. The dynamic tree cut were modules divided according to the clustering results, and merged dynamic were modules that were merged with a similar representation pattern based on module similarity. The major tree branches constitute 15 merged modules labeled with different colors. **(B)** Module-stage relationships. Each row corresponds to a module, and each column represents a specific stage. The color of each cell at the row-column intersection indicates the correlation coefficient between a module and a stage. A high degree of correlation between a specific module and stage is indicated by red. **(C)** Eigengene expression profile of each module. The y-axis indicates the value of the module eigengene; the x-axis indicates the sampled post-germination stage (1–5).

We performed WGCNA to find the co-expression network of genes specifically expressed in the *M. pasquieri* post-germination stages from a comprehensive network perspective. The co-expression network was constructed with all the DEGs at different stages. The genes with similar expression patterns were clustered into the same modules, and different modules were distinguished by color, as shown in Figure 5A. Finally, 14 different merged modules were identified, and the subsequent analysis was carried out according to the merged modules. According to correlation analysis, these modules corresponded to a specific distribution pattern in the post-germination stages (Figure 5B). Compared with other modules, such as grey 60, the genes of this module displayed the highest correlation with stage 4 (*p* = 0.004, *r* = 0.69), while the genes in the purple module showed the highest correlation with stage 5 (*p* = 3 × 10^6^, *r* = 0.91). The genes in the pink, magenta, and light-yellow modules were most associated with stages 1 (*p* = 6 × 10^6^, *r* = 0.9), 2 (*p* = 0.04, *r* = 0.53), and 3 (*p* = 0.02, *r* = 0.58), respectively.

The ME values are the principal component of a gene module, which represents the gene expression profile of each module. The eigengene expression profiles of 11 modules were analyzed, as shown in Figure 5C. MEs of the pink, green-yellow, and light cyan modules had higher expression levels during stage 1, whereas MEs of the magenta and grey 60 modules were expressed at higher levels during stages 2 stage 4. MEs of the light yellow and tan modules had the highest expression during stage 3. During stage 5, MEs of the dark red, purple, light green, and red modules were expressed at higher levels.

The categories of enriched pathways and their mobilization trends in each module were determined for the purpose of understanding the changes of biological processes in different modules. By comparing the number of background genes, genes in the magenta module were significantly enriched in six categories: “glycolysis/gluconeogenesis,” “glutathione metabolism,” “nicotinate and nicotinamide metabolism,” “biosynthesis of secondary metabolites,” “phenylpropanoid biosynthesis,” and “alpha-Linolenic acid metabolism.” The light yellow module was significantly correlated with stage 3, with “RNA polymerase,” “tropane, piperidine, and pyridine alkaloid biosynthesis,” and “pyrimidine metabolism” being significantly enriched. The purple module was significantly associated with stage 5, with 13 significantly enriched categories identified (Supplementary Table 5). The number of enriched categories varied in the other modules.

**Figure 6:**
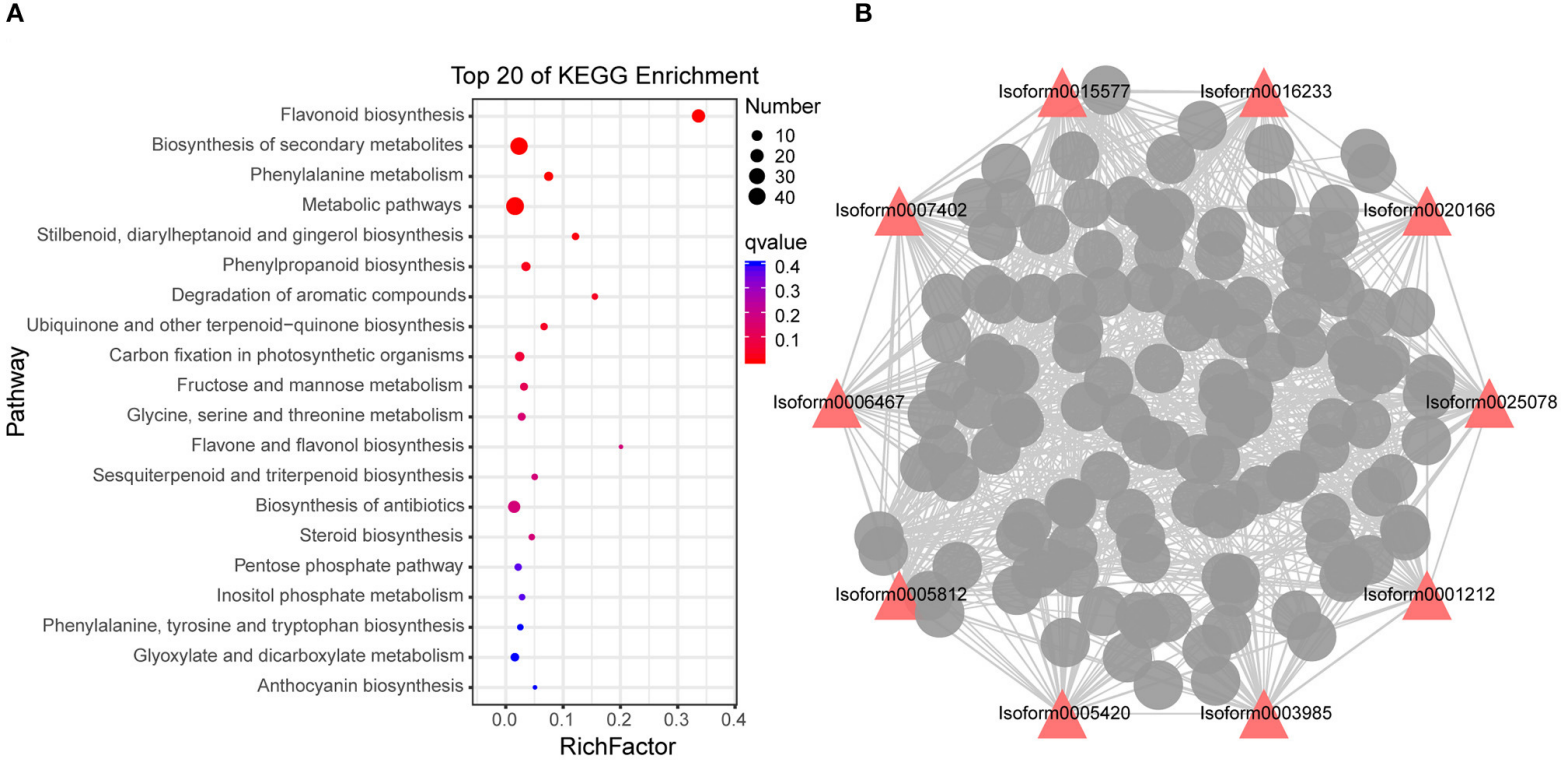
Co-expression network analysis of grey 60 module. **(A)** Enriched Kyoto Encyclopedia of Genes and Genomes (KEGG pathways) of the grey 60 module genes. **(B)** Correlation network of genes in the grey 60 module (stage 4). *q*-value is a *P*-value that has been adjusted for the false discovery rate (FDR). A lower *q*-value indicates that a lower percentage of significant results will be false positives. Rich factor represents the degree of enrichment of genes under the designated pathway term, the greater the value of the rich factor, the greater the degree of pathway enrichment. Red triangles indicate the top 10 hub genes, the wider the edge, the greater the weight between the two genes.

Among the 14 modules, we found that the number of flavonoids accounted for the largest proportion among the DAMs in this stage from the metabolome results; thus, the following main analysis was performed on the grey 60 module. “Flavonoid biosynthesis,” “biosynthesis of secondary metabolites” and “phenylalanine metabolism” were significantly enriched in grey 60 module genes (Figure 6A), which further proved that “flavonoid biosynthesis” was active in stage 4. WGCNA is also available for constructing gene co-expression networks, where each node represents a gene, and the connecting lines between nodes are called edges, which represent the co-expression of related genes. The node with the highest connectivity, named hub gene, may play a vital role in the different modules. The grey 60 module network is shown in Figure 6B, and the top 10 hub genes are identified by red triangles, among which Isoform0005812, Isoform0003985, and Isoform0006467 were assigned to “flavonoid biosynthesis” (ko00941) (Supplementary Table 6). This indicated that hub genes were mainly involved in “flavonoid biosynthesis,” except for “metabolic pathways” and “biosynthesis of secondary metabolites.”

### Transcript-Metabolite Correlation Network

**Figure 7:**
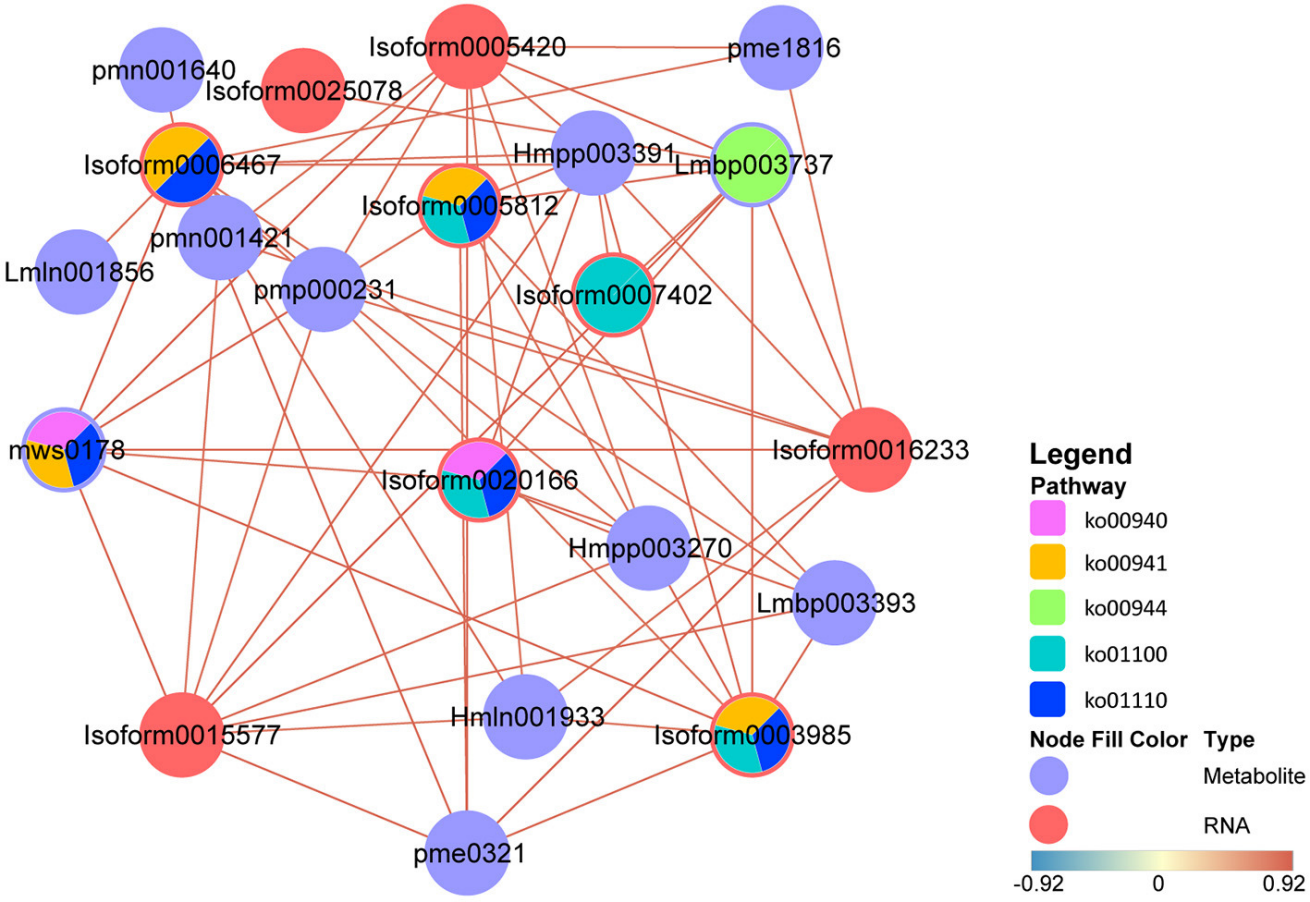
Transcript-metabolite Pearson's correlation network representing DAMs and DEGs involved in *M. pasquieri*. The gene-metabolite pairs were connected within the network by edges. Red nodes represent genes, and purple nodes represent metabolites. The edges between nodes represent correlation. The color bar below indicates red edges represent positive correlations, and blue edges represent negative correlations. Only correlation pairs with a correlation coefficient > 0.8 were included in the network. The pie chart on the nodes shows part of pathways involved in DAM and DEG. DAMs, differentially accumulated metabolites; DEGs differentially expressed genes. Lmbp003393, quercetin-3-O-α-L-rhamnopyranoside; pme0321, kaempferol 7-O-rhamnoside; pmn001640, myricetin 3-α-L-arabinofuranoside; Lmbp003737, kaempferol-3-O-rhamnoside; Hmln001933, myricetin 3-O-galactoside; Hmpp003391, luteolin-3′-O-β-D-glucoside; Hmpp003270, luteolin-4′-O-β-D-glucoside; mws0178, chlorogenic acid; pmn001421, 3-O-(E)-p-Coumaroyl quinic acid; pmp000231, trans-3-O-p-coumaric quinic acid; pme1816, neochlorogenic acid; Lmln001856, 5,7-dihydroxy-4-oxo-2-(3,4,5-trihydroxyphenyl)-4H-chromen-3-yl-β-D-allopyranoside.

To simulate the regulatory properties of DAMs and DEGs, a subnetwork was constructed for the top 10 hub genes to determine transcript-metabolite correlations. Pearson's correlation tests were carried out between relative quantitative changes of metabolites and related transcripts, and we set correlation coefficient > 0.8 as cut-off in the analysis. Meanwhile, the pathways involved in DAMs and DEGs were shown by the pie chart; it can be found that except for “metabolic pathways,” DAMs and DEGs were most involved in “flavonoid biosynthesis” (Figure 7). Not only that, in these DAMs, four flavonols and three flavonoids were found. These results indicated that the top 10 hub genes were highly correlated with their corresponding metabolites involved in “flavonoid biosynthesis,” and many flavonols were identified in this process, which reconfirmed the large accumulation of flavonols and their special importance during stage 4. The authenticity and accuracy of the metabolic analysis were validated by transcriptome data.

### Flavonol Biosynthesis Pathway

**Figure 8:**
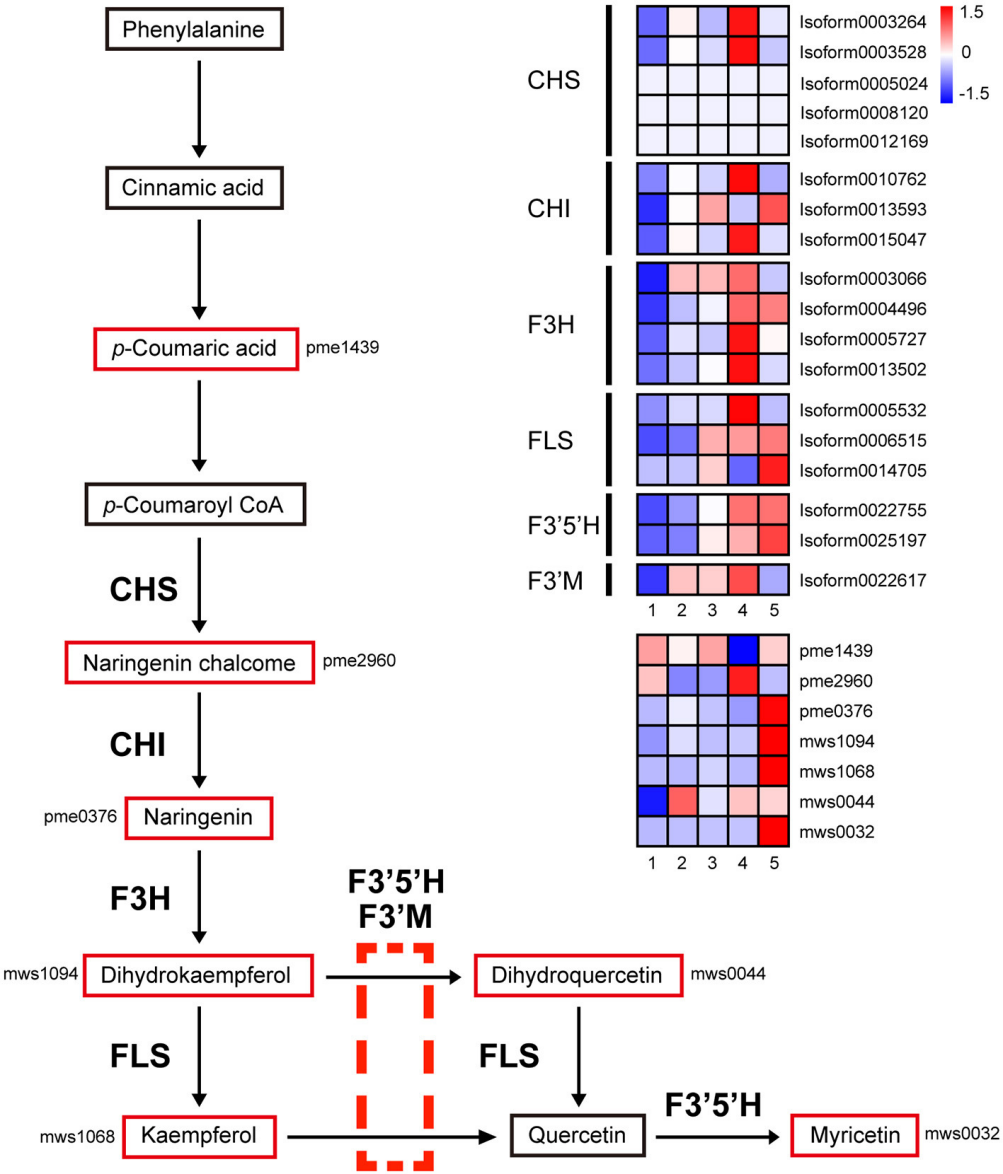
Transcript and metabolic profiling of genes in the flavonoid and flavonol biosynthetic pathways in *M. pasquieri*. The heatmap is drawn according to the average expression levels of related biosynthetic genes and relative accumulation levels of related metabolites. A color bar is presented at the top right, and the colors from blue to red indicate low to high expression and accumulation. CHS, chalcone synthase; CHI, chalcone isomerase; F3H, naringenin 3-dioxygenase; FLS, flavonol synthase; F3′5′H, flavonoid 3′,5′-hydroxylase; F3′M, flavonoid 3′-monooxygenase.

The flavonol biosynthesis pathway involves three pathways: “phenylpropanoid biosynthesis,” “flavonoid biosynthesis,” and “flavone and flavonol biosynthesis.” As shown in Figure 8, after a series of conversions, phenylalanine is converted to *p*-Coumaroyl coenzyme A (CoA) through a series of enzymes in the “phenylpropanoid biosynthesis” pathway. Since then, many genes and metabolites of the latter two pathways were upregulated in stage 4, such as chalcone synthase (CHS, five DEGs), chalcone isomerase (CHI, three DEGs), naringenin 3-dioxygenase (F3H, four DEGs), flavonol synthase (FLS, three DEGs), flavonoid 3′,5′-hydroxylase (F3′5′H, 2 DEGs), and flavonoid 3′-monooxygenase (F3′M, 1 DEG).

Most of these genes were significantly upregulated in stage 4 (Figure 8). The expression levels of two CHS genes, Isoform0003264, and Isoform0003528, were 2.54 and 2.44, respectively, times higher in stage 4 than in stage 3, indicating the high accumulation of naringenin chalcone in stage 4. Naringenin was produced from naringenin chalcone, catalyzed by CHI (Isoform0010762 and Isoform0015047; 3.91- and 2.88-fold upregulation in stage 4; however, Isoform0013593 downregulated by about one time but upregulated again in stage 5), and naringenin was not upregulated in stage 4, but was significantly accumulated in stage 5. Naringenin 3-dioxygenase catalyzes the conversion of naringenin into dihydrokaempferol, and its encoding gene F3H, Isoform0003066, Isoform0004496, Isoform0005727, and Isoform0013502 were upregulated 1.24, 1.89, 3.73, and 2.67 times in stage 4, respectively. The content of dihydrokaempferol was significantly accumulated in stage 5; the upregulation was not significant in stage 4. FLS catalyzes the conversion of dihydrokaempferol into kaempferol, and two genes (Isoform0005532 and Isoform0006515) were upregulated in stage 4; however, the content of kaempferol was also significantly increased in stage 5. Meanwhile, F3′5′H and F3′M can catalyze the conversion of dihydrokaempferol and kaempferol into dihydroquercetin and quercetin, respectively. In stage 4, there were two F3′5′H genes (Isoform0022755 and Isoform0025197) and one F3′M gene (Isoform0022617) that were up-regulated. Quercetin can also be obtained by the FLS catalytic conversion of dihydroquercetin, and quercetin can be further catalyzed by F3′5′H to myricetin.

### Plant Hormone Signal Transduction Pathway

**Figure 9:**
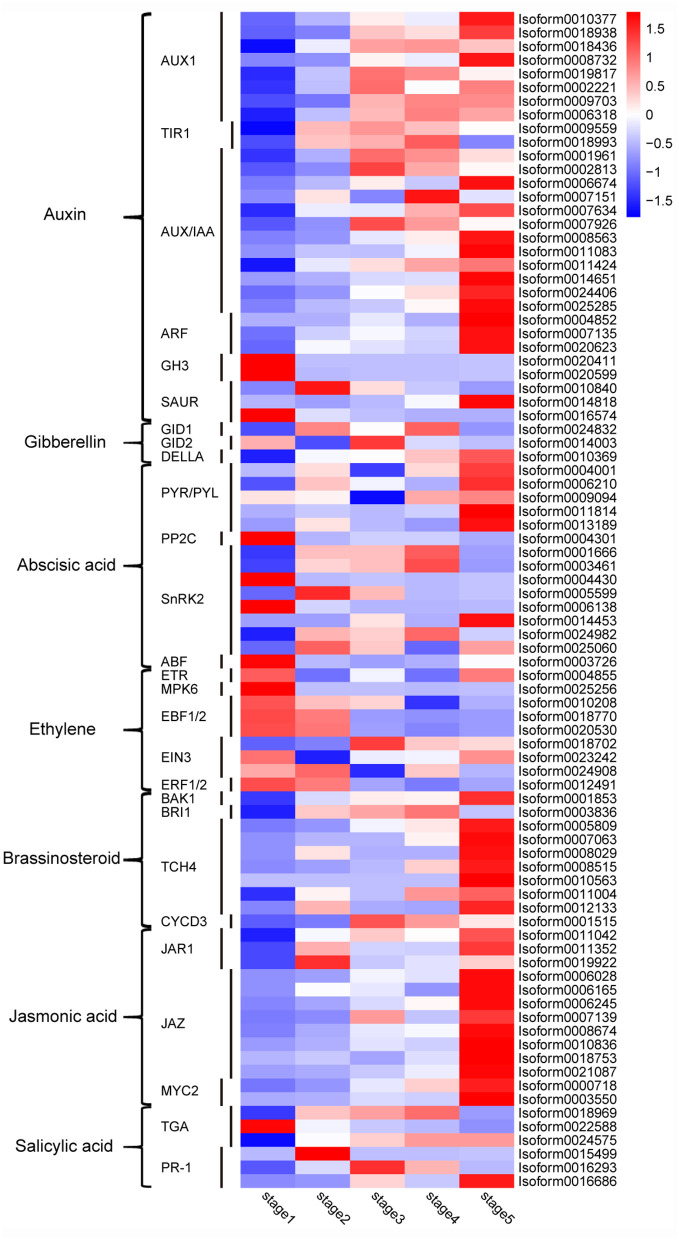
Heatmap of expression of DEGs in the plant hormone signal transduction pathway. A color bar is presented at the top right, and the colors from blue to red indicate low to high expression. AUX1, auxin transporter protein 1; TIR1, transport inhibitor response1; AUX/IAA, auxin/indole-3-acetic acid; ARF, auxin response factor; GH3, gretchenhagen 3; SAUR, small auxin upregulated RNA; GID1, GA-insensitive dwarf mutant 1; GID2, GA-insensitive dwarf mutant 2; PYR/PYL, pyrabactin resistance/PYR-like; PP2C, protein phosphatase 2 C; SnRK2, sucrose non-fermenting 1-related protein kinases subfamily 2; ABF, ABRE-binding factor; ETR, ethylene receptor; MPK6, mitogen-activated protein kinase 6; EIN3, ethylene insensitive 3; EBF1/2, EIN3-binding F-BOX 1 and 2; ERF1/2, ethylene-responsive transcription factors 1 and 2; BAK1, brassinosteroid-insensitive 1-associated receptor kinase 1; BRI1, brassinosteroid-insensitive 1; TCH4, xyloglucosyl transferase TCH4; CYCD3, cyclin D3; JAR1, jasmonic acid-resistant 1; JAZ, jasmonate ZIM domain-containing protein; MYC2, transcription factor MYC2; TGA, transcription factor TGA; PR-1, pathogenesis-related protein 1.

Plant hormones, such as auxin, gibberellin (GA), cytokinine, and abscisic acid (ABA), are closely related to post-germination growth. However, 349 genes were found and involved in the “plant hormone signal transduction” pathway; but only one metabolite, salicylic acid, was found in the whole pathway (Supplementary Figure 7). In order to study the genes related to plant hormones further, 86 DEGs were selected from the 349 genes, and their expression levels were analyzed with a heatmap. As shown in Figure 9, there are 30 auxin-related DEGs, of which most auxin transporter protein 1 (AUX1) genes are upregulated in stages 3–5; the expression of transport inhibitor response1 (TIR1) genes is upregulated in stages 2-4; most of the auxin/indole-3-acetic acid (AUX/IAA) and auxin response factor (ARF) genes are upregulated in stage 5; most of the gretchenhagen 3 (GH3) and small auxin-upregulated RNA (SAUR) genes are upregulated in the first two stages. Only three DEGs were associated with GA, namely, GA-insensitive dwarf mutant 1 (GID1), GA-insensitive dwarf mutant 2 (GID2), and DELLA. Among them, GID1 was upregulated in stages 2 and 4, GID2 was upregulated in stages 1 and 3, and DELLA was upregulated in stages 4 and 5. There were 15 ABA-related DEGs, among which all pyrabactin resistance/PYR-like (PYR/PYL) genes were upregulated in stage 5; however, most of the protein phosphatase 2 C (PP2C), sucrose non-fermenting 1-related protein kinases subfamily 2 (SnRK2), and ABRE-binding factor (ABF) genes were upregulated in the first three stages. Most ethylene-related DEGs, such as ethylene receptor (ETR), mitogen-activated protein kinase 6 (MPK6), ethylene insensitive3 (EIN3), EIN3-binding F-BOX1 and 2 (EBF1/2), and ethylene-responsive transcription factors 1 and 2 (ERF1/2), were upregulated in stages 1 and 2. Most of the brassinosteroid insensitive 1-associated receptor kinase 1 (BAK1), brassinosteroid-insensitive 1 (BRI1), xyloglucosyl transferase TCH4 (TCH4), and cyclin D3 (CYCD3) genes related to brassinosteroid, and jasmonic acid resistant1 (JAR1), jasmonate ZIM domain-containing protein (JAZ), and transcription factor MYC2 (MYC2) genes related to jasmonic acid were upregulated in stage 5. However, DEGs related to salicylic acid, transcription factor TGA (TGA) and pathogenesis-related protein 1 (PR-1), were upregulated in the different stages. The cytokinine-related genes changed indistinctively in stages 1–5, so they were not included in this analysis.

### Flavonol Affects Polar Auxin Transport

**Figure 10:**
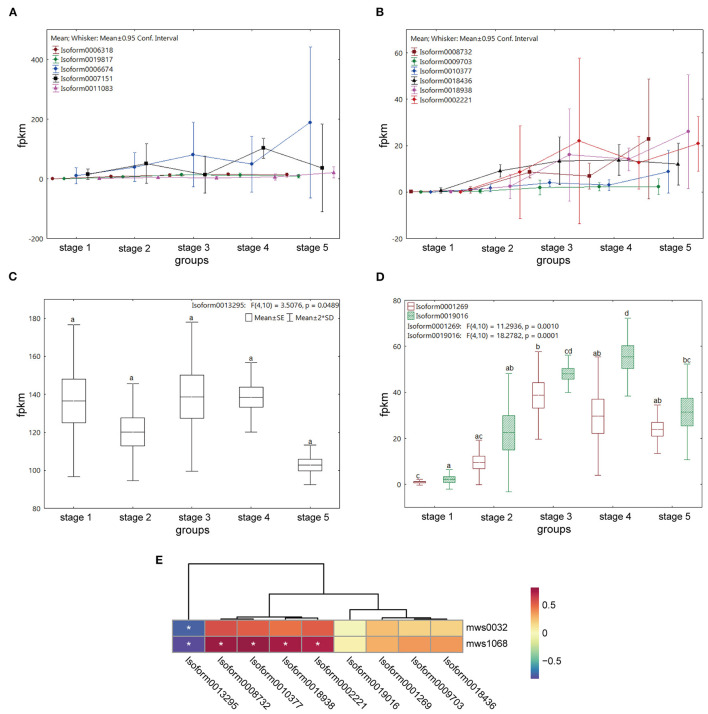
Expression of the genes encoding polar auxin transport carriers at the five stages and its correlation with flavonol contents during stages 4 and 5 in *M. pasquieri*. **(A)** Expression of five genes encoding AUX. **(B)** Expression of six genes encoding LAX. **(C)** Expression of the gene encoding PIN4. **(D)** Expression of two genes encoding ABCB1. AUX/LAX, Auxin1/Like-AUX1; PIN4, pin-formed protein4; ABCB1, ATP-binding cassette subfamily B1. **(E)** Heatmap of correlation between genes encoding polar auxin transport carriers and flavonol contents during stages 4 and 5. In the color bar, red represents a positive correlation, and blue represents a negative correlation. *p < 0.05.

In all, the 349 genes were upregulated and involved in the “plant hormone signal transduction” pathway; however, only one metabolite, salicylic acid, was found in the whole pathway (Supplementary Figure 7). In many previous studies, flavonols, a flavonoid subgroup, have been found to significantly affect the polar transport of auxin and can be used as plant transport inhibitors (Buer et al., 2010). Polar auxin transport carrier was mainly completed by the influx carrier Auxin1/Like-AUX1 (AUX/LAX), efflux carrier ATP-binding cassette subfamily Bs/P-glycoprotein (ABCBs/PGP), and the pin-formed (PIN) protein family (Teale and Palme, 2018). The gene expression levels of the above five encoding proteins at each stage were analyzed, and no gene encoding PGP was found in *M. pasquieri* transcripts in all the stages. Five genes were encoding AUX, among which Isoform0006674 and Isoform0007151 were significantly down- and upregulated in stage 4, respectively, while the other genes had no significant changes (Figure 10A). Among the six genes encoding LAX, Isoform0008732, Isoform0010377, Isoform0018938, and Isoform0002221 are all downregulated in stage 4 and upregulated in stage 5 (Figure 10B). Seven and 13 genes were found encoding PIN and ABCB, respectively, but only one gene (Isoform0013295) was found encoding pin-formed protein4 (PIN4), and two genes were found encoding ATP-binding cassette subfamily B1 (ABCB1, Isoform0001269, and Isoform0019016). Among them, no significant expression difference of Isoform0013295 in each stage had been found. The expression level of Isoform0001269 was downregulated in stages 4 and 5, but not significantly, while Isoform0019016 had a higher expression in stage 4 (Figures 10C,D).

To further investigate the relationship between flavonols and gene expression of these polar auxin transport carriers, a correlation heatmap was constructed (Figure 10E). The result showed that the contents of kaempferol (mws1068) and myricetin (mws0032) were negatively and significantly correlated with the expression level of the PIN4 encoding gene (Isoform0013295), respectively, while kaempferol was positively and significantly associated with the expression levels of four genes encoding LAX (Isoform0008732, Isoform0010377, Isoform0018938, and Isoform0002221), respectively. Additionally, there was no obvious correlation between the content of myricetin and the expression levels of genes encoding LAX, or between the contents of kaempferol and myricetin and the expression levels of ABCB1 encoding genes.

### Validating Gene Expression Patterns by RT-qPCR

**Figure 11:**
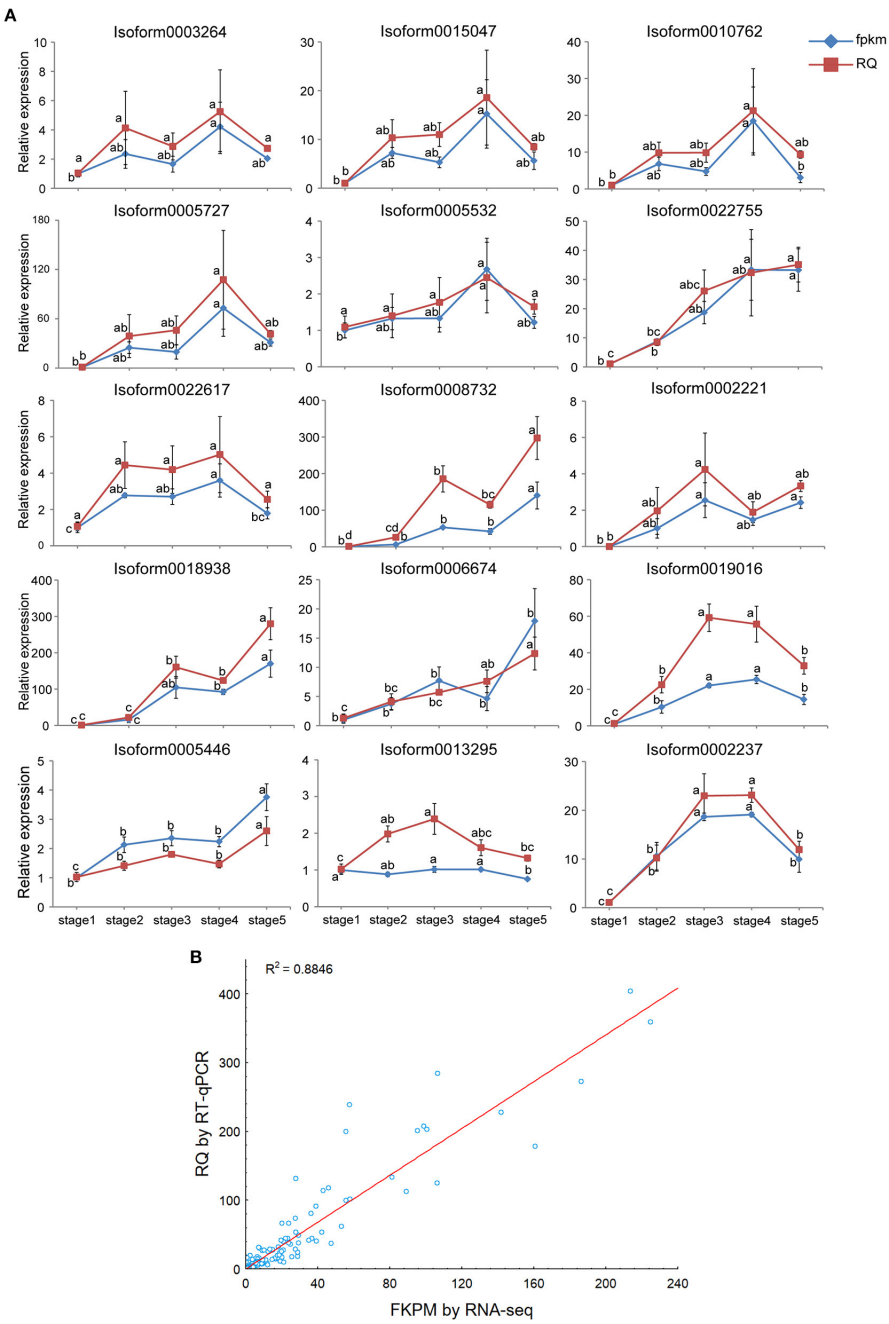
Real-time quantitative polymerase chain reaction (RT-qPCR) validation of 15 structural genes. **(A)** Expression patterns of the 15 genes involved in the flavonol biosynthesis and polar auxin transport in five post-germination stages of *M. pasquieri* (from stages 1 to 5). Each column represents an average of three biological replicates, with standard errors indicated by vertical bars. Values with a different accompanying letter are statistically significantly different according to Duncan's multiple range test at *p* < 0.05. **(B)** Correlation of the expression levels of 15 selected genes measured by RNA-seq and RT-qPCR.

To identify the actual expression patterns of key DEGs involved in the flavonol biosynthesis pathway and polar auxin transport, RT-qPCR was performed to validate the seven DEGs encoding CHS (1), CHI (2), F3H (1), FLS (1), F3′5′H (1), and F3′M (1) from the flavonol biosynthesis pathway and eight DEGs encoding LAX (3), AUX (1), ABCB (2), and PIN (2) belong to the polar auxin transport carriers in the five post-germination stages of *M. pasquieri*. The results of the RT-qPCR were consistent with the RNA-Seq data (Figure 11A) and indicated that the qRT-PCR and RNA-Seq data were highly correlated and presented consistency in the upregulation and downregulation of DEG expression (*r*^2^ = 0.8846) (Figure 11B). These results indicated that the RNA-Seq data were reliable.

## Discussion

### Differentially Accumulated Metabolites (DAMs) Specifically Involved in Different Post-Germination Stages

We detected a total of 497 metabolites in the five post-germination stages of *M. pasquieri*, including 34 substances and their derivatives (Supplementary Table 2), among which phenolic acids (82, 16.5%), amino acids and derivatives (76, 15.3%), organic acids (39, 7.8%), nucleotides and derivatives (38, 7.6%) and flavonols (32, 6.4%) accounted for the largest proportion (Figure 2B). However, Wang et al. (2020) only identified 57 metabolites in mung bean by untargeted metabolome analysis performing gas chromatography-mass spectrometry (GC-MS), and most of the metabolites were sugar metabolism compounds, amino acid metabolism compounds, tricarboxylic acid (TCA), and other organic acid metabolism compounds. Seven hundred thirty metabolites were detected in the germination and post-germination growth stages of the two varieties of rice by widely targeted metabolome, including 32 substances and their derivatives, among which flavone (74, 10.1%), organic acids (67, 9.2%), amino acid derivatives (60, 8.2%), nucleotide and its derivates (57, 7.8%), and flavone C-glycosides (44, 6.0%) accounted for the largest proportion (Yang et al., 2020). This indicated that the widely targeted metabolome method could identify more metabolites than the untargeted metabolome method, and the metabolites of rice were far more than that of *M. pasquieri*, which may be related to the different species and germination stages.

We found that the maximum number of DAM was observed in stage 5, which may be because the seedlings in the nine-leaf stage have begun to use photosynthesis for energy supply, participate in a variety of metabolic pathways, and are active in the synthesis and degradation of various metabolites. Interestingly, except for the highest proportion of flavonoids in the DAMs for stage 4 vs. stage 1, the DAMs of the other groups had the largest proportion of amino acids and derivatives (Figure 3), indicating the largest change in flavonoid substances in stage 4. In addition, the contents of organic acids, phenolic acids, tannins, and lipids also changed significantly in each stage.

In the analysis of DAMs in the five stages, it was found that compared with stage 1, most of the lipids were significantly accumulated in stages 2 and 5, the majority of tannins were significantly accumulated in stages 2 and 3, terpenoids were mainly significantly accumulated in stage 3, and most of the phenolic acids were significantly accumulated in stages 4 and 5. Most of flavonoids were increased to different degrees in the five stages (Supplementary Table 3). However, in a study on poplar post-germination, lipids were found decreased during the hypocotyl elongation stage and increased in the seedling stage, suggesting that this may be related to the increased demand for lipids in poplar seeds (Qu et al., 2019a). This result was contrary to those of this study, which may be because of the fact that *M. pasquieri* is a woody oil tree, whose seeds have a high oil content, so its lipids metabolism was still active in stage 2. Interestingly, in both poplar and mung bean studies, amino acids and derivatives were obviously upregulated from the hypocotyl elongation stage, which may be related to the decomposition of storage proteins (Qu et al., 2019a; Wang et al., 2020). However, in this study, most of the amino acids and derivatives were significantly downregulated in stages 2–5 (Supplementary Table 3), which may be caused by the requirement of high levels of protein synthesis in the post-germination of *M. pasquieri*, but more in-depth studies are needed. These results indicate that although poplar and *M. pasquieri* are both woody plants, they belong to Sapotaceae and Salicaceae, respectively, and there are significant differences in the changes in metabolites in post-germination growth. In addition, most tannins were significantly upregulated in stages 2 and 3, which was consistent with the previously reported tannins in *M. pasquieri* (Kan et al., 2020), indicating that tannins had begun to accumulate in the early post-germination stage. The terpenoids accumulated significantly in stage 3 and were all triterpene (Supplementary Table 3). Triterpene is not only important for the formation of saponins and resins in plants; it is also related to defense function (Kemen et al., 2014), indicating that terpenoid metabolism became active in stage 3, possibly in preparation for environmental adaptation. Moreover, phenolic acids, which act as signal molecule and defense plant herbivores and pathogens, accumulated significantly in later two stages (Mandal et al., 2010). These indicated that secondary metabolites had been gradually active since stage 3 in *M. pasquieri*. All the DAMs that were upregulated in each stage had flavonoids, indicating that they played a vital role in the post-germination growth of *M. pasquieri*.

### Transcriptome Analysis of Post-germination Stages in *M. pasquieri*

This study found that the mapped rate of transcripts from the five post-germination stages to the reference sequence of *M. pasquieri* was 68.8–76.18%, which was lower than that of other studies. For example, 78.7–83.9% of the reads were mapped on the mung bean reference genome (Wang et al., 2020). The average rates of mapped transcripts for the *indica* rice and *japonica* rice in the Nipponbare reference genome were 80.94 and 80.27%, respectively (Yang et al., 2020). This is because *M. pasquieri* lacks genomic information and only full-length transcriptome sequences are used as reference sequences, while the third-generation sequencing uses mixed samples, which may filter some low-quality reads during assembly, and the transcript length is longer, leading to a relatively lower mapped rate with the single RNA-Seq samples. Transcripts of stages 2–5 were compared with those of stage 1 to obtain DEGs for WGCNA.

Weighted gene co-expression network analysis is a biological tool that provides an effective way to analyze correlations between gene expression levels from complex RNA-Seq data at different developmental stages, treatments, and time courses (Yuan et al., 2018). The WGCNA results showed that the magenta and light yellow modules were mostly correlated with stages 2 (hypocotyl elongation) and 3 (epicotyl elongation). Genes of the magenta module were mainly enriched in “glycolysis/gluconeogenesis,” “glutathione metabolism,” “nicotinate and nicotinamide metabolism,” “biosynthesis of secondary metabolites,” and “phenylpropanoid biosynthesis” (Supplementary Table 5), which were consistent with the enrichment pathways of the rice hypocotyl elongation stage (Yang et al., 2020). However, in other studies, the genes during this stage were significantly enriched in starch and sucrose metabolism, glycolysis, TCA cycle, biosynthesis of amino acids, and plant hormone signal transduction pathway (Sreenivasulu et al., 2008; Wang et al., 2020), where glycolysis and TCA cycle provide the required energy for subsequent growth. The light yellow module was significantly enriched in “RNA polymerase,” “tropane, piperidine, and pyridine alkaloid biosynthesis,” and “pyrimidine metabolism” pathways (Supplementary Table 5), which was completely different from previous reports that DEGs were significantly enriched in carbohydrate synthesis, biosynthesis of amino acids, and plant hormone signal transduction during the epicotyl elongation stage in mung bean (Wang et al., 2020). This may be caused by the specific growth characteristics of woody plants and herbaceous plants, and the bias of different growth stages in post-germination, leading to different enrichment pathways. The purple module had the strongest correlation with stage 5 (nine-leaf), which was mainly enriched in photosynthesis, photosynthesis-related metabolic pathways, nitrogen metabolism, carbon metabolism, and amino acid metabolism pathways, which was consistent with previous studies (An and Lin, 2011; Qu et al., 2019b). It is worth noting that the grey 60 module had the strongest correlation with stage 4 (two-leaf), significantly enriched in secondary metabolites biosynthesis pathways, such as flavonoid biosynthesis, phenylalanine metabolism, stilbenoid, diarylheptanoid, and gingerol biosynthesis (Supplementary Table 5), and the key genes with the high connectivity within the module were also mainly involved in flavonoid biosynthesis (Supplementary Table 6). However, using the same WGCNA method, the study on poplar germination in different stages showed that the module key genes of the highest correlation with the two-leaf stage were mainly involved in CHO, cell wall, lipids metabolism, and PS (Qu et al., 2019b); this discrepancy may be the result of differences between the species being studied. We speculate that the energy reserve and secondary metabolite synthesis significantly changed in stage 2 of *M. pasquieri*, that photosynthesis significantly changed in stage 5, and that the pathway significantly enriched in stage 3 still needs to be further studied. The significant changes in secondary metabolites, such as flavonoid biosynthesis in stage 4, might be the potential reason for the slow growth of *M. pasquieri* seedlings in the early culture process.

Post-germination growth is regulated by the synergistic interaction of various endogenous plant hormones (Miransari and Smith, 2014). However, the WGCNA results showed that the genes in the modules of interest were not significantly enriched in the “plant hormone signal transduction” pathway (Supplementary Table 5), and that only one metabolite, salicylic acid, was detected in this pathway (Supplementary Figure 7). In order to further explore these results, the expression levels of plant hormone-related genes in the pathway were analyzed (Figure 9). Auxin is known to promote cell elongation and plays an important role in post-germination growth (Pacifici et al., 2015). Most of the DEGs related to auxin signal transduction, such as AUX1, TIR1, AUX/IAA, and ARF, were upregulated in stage 3–5, indicating that the auxin signal transduction process was active from epicotyl elongation and promoted the growth of *M. pasquieri*. GA promotes growth mainly by stimulating radicle elongation (Romero-Rodriguez et al., 2018; Song et al., 2019); we have also found that its related DEGs were upregulated in the different stages of *M. pasquieri* (Figure 9). In addition to inhibiting seed germination, ABA is also involved in regulating stomatal closure and reducing evapotranspiration (Jin et al., 2013). In this study, most ABA-related genes, such as PP2C, SnRK2, and ABF, were upregulated in the first three stages, while PYR/PYL genes were upregulated in stage 5, which may be related to different environmental stress conditions in the different stages. Except for auxin, GA, and ABA, other plant hormones like ethylene, brassinosteroid, jasmonic acid, and salicylic acid were also important in the growth of post-germination. Ethylene not only promotes fruit ripening and senescence, in other studies, it has also been reported to promote seed germination and growth (Wang et al., 2020). Based on the results of this study, it was found that most of the ethylene-related DEGs were upregulated in stages 1 and 2 (Figure 9), which may be related to the promotion of growth in the early post-germination stages. Studies have reported that brassinosteroid mainly promotes cell division and cell elongation (Pacifici et al., 2015), and that jasmonic acid is mainly involved in stress response (Zhao et al., 2019). Most of the DEGs related to brassinosteroid and jasmonic acid were increased in stage 5 (Figure 9), indicating that other than growth promotion, there may have been anti-stress involved in stage 5. Salicylic acid, known for its disease resistance response, is also involved in the regulation of physiological and chemical processes in plants (Matic et al., 2016). Salicylic acid was also the only plant hormone detected in this study, and was significantly accumulated in all the stages, while its related DEGs upregulated in the different stages (Figure 9), indicating that salicylic acid metabolism was activated from stage 1, which may be actively involved in the regulation of disease resistance process.

### Flavonol Affects Polar Auxin Transport

Flavonoids are secondary metabolites widely distributed in plants with diverse physiological functions (Taylor and Grotewold, 2005; D'Amelia et al., 2018), which play important roles such as ultraviolet photo-damage protection (Biever and Gardner, 2016), defense against pathogens and pests (Zhao et al., 2020), stress response (Gu et al., 2020), pollen and pollen tube formation (Zhang et al., 2020b), and auxin transport regulation (Ramos et al., 2016). Flavonols, a subgroup of flavonoids, regulate auxin transport and auxin-dependent physiological processes, and act as an auxin transport inhibitor (Pollastri and Tattini, 2011; Brunetti et al., 2018). Many studies have reported that flavonols, such as kaempferol, quercetin, isorhamnetin, and myricetin, are distributed in plants (Zhang et al., 2020a). In addition, naringenin, as a precursor of flavonols, has strong inhibitory effects on *Arabidopsis* seed germination and main root growth of seedlings (Hernández and Munné-Bosch, 2012). The polar auxin transport carriers are mainly completed by influx carrier AUX/LAX, efflux carrier ABCBs/PGP, and PIN protein families (Mohanta et al., 2018). However, flavonol can affect ABCBs/PGP and PIN carriers (Teale and Palme, 2018), and can also be converted to the glycosyl form under the action of UDP-glycosyltransferase to affect auxin transport carriers (Kuhn et al., 2016; Yin et al., 2017). The expression levels of pin-formed protein1 (PIN1) were found to be decreased in the *tt4* mutant (without flavonoid synthesis), which affects PINs protein aggregation and circulation, and enhances auxin transport; while the high content of quercetin and kaempferol in the *tt7* mutant (excessive accumulation of kaempferol) inhibits auxin transport (Peer et al., 2004; Buer et al., 2013).

At present, the chemosmosis hypothesis is widely accepted in the study on polar auxin transport, that is, auxin can form ionized IAAH^+^ outside plastid (pH = 5.5), and the lipophilicity of ionized IAAH^+^ can be diffused or form the ion with influx carrier AUX/LAX flow into the cytoplasm. After entering the cell, auxin is deionized in the neutral cytoplasm and can only be transported out of the cell by efflux carrier ABCBs/PGP and PIN, among which ABCBs are mainly responsible for long-distance non-directional transport, while PIN is mainly involved in the intercellular directional transport of auxin (Mohanta et al., 2018). There have been many reports that flavonol is considered an auxin transport inhibitor (Ramos et al., 2016; Brunetti et al., 2018). The results of this study found that most of the genes that participated in the flavonol biosynthesis pathway were obviously upregulated during stage 4, and some were upregulated during stage 5, but most of the metabolites involved in the pathway were apparently accumulated only in stage 5, such as the final flavonols, kaempferol, and myricetin (Figure 8). It might be that the flavonol biosynthesis pathway in stage 4 significantly enriched (Figure 6A), which was consistent with the WGCNA results. In this stage, most genes began to be upregulated to carry out transcription and translation and eventually become metabolites, which took some time. Therefore, most of the metabolites accumulated significantly in stage 4, indicating that the changes in the metabolites seemed to be driven by increased transcript levels. In addition, in the metabolome data, it can be found that flavonoid accounted for the largest proportion of DAMs in stage 4 (Figure 3C), indicating that flavonoid changed significantly in stage 4, but that it still needed a certain time for the accumulation of substances.

PGPs belong to the ABCB transporter family and can hydrolyze ATP to transport substrates (Peer and Murphy, 2007). Some studies have shown that flavonols can inhibit P-glycoproteins (PGP) transport auxin by affecting the expression pattern of the PGP gene and its subcellular localization (Lewis et al., 2007). In this study, no transcript encoding PGP was found, so it was speculated that the accumulation of flavonols may directly inhibit the expression of PGP encoding genes, which needed further studies. PIN plays an essential part in regulating auxin distribution in plants and affecting plant development (Kreček et al., 2009). Studies have shown that the PIN protein mediates auxin effluxion (Zhou and Luo, 2018; Yang et al., 2019a). Flavonols can indirectly affect the gene expression and subcellular localization of PIN1 and pin-formed protein2 (PIN2) (Peer and Murphy, 2007; Santelia et al., 2008). In a study on *Arabidopsis rol1-2* mutants, it was found that flavonols changed the polar localization of PIN2, thus affecting auxin transport (Kuhn et al., 2017). It has also been shown that a flavonol interacted with the protein complex of PIN1, making it more stable and less able to mediate cellular auxin efflux (Teale et al., 2021). Moreover, some research studies have found that flavonols directly affected PIN4 expression and subcellular localization, and changed the intercellular concentration of auxin (Peer and Murphy, 2007). In this study, seven genes encoding PIN were found, but only one gene encoding PIN4 (Isoform0013295) was downregulated in stage 5, but not significant (Figure 10C). Further correlation analysis revealed that the contents of kaempferol and myricetin were significantly negatively correlated with Isoform0013295 in stages 4 and 5, respectively (Figure 10E), which suggested that flavonols might inhibit PIN4 encoding gene expression to some extent. Some studies suggest that ATP-binding cassette subfamily B19 (ABCB19), rather than PIN1, was the target of 1-naphthylphthalamic acid (NPA)-mediated polar auxin transport inhibition, and that flavonols can compete with NPA for binding sites (Geisler et al., 2016). In addition, flavonols can bind and inhibit the transport proteins ABCB1 and ABCB19, and interfere with their interaction with FK506-binding protein TWISTED DWARF1 (FKBP42/TWD1), thus inhibiting the transport of auxins, among which quercetin has the best effect (Muday et al., 2006; Geisler et al., 2016), but no studies have proved that flavonols directly inhibit the expression of ABCB encoding genes. A total of 13 ABCB encoding genes were found in this study, two of which were encoding ABCB1 (Isoform0001269 and Isoform0019016), but no ABCB19 encoding gene was found. Isoform0001269 was downregulated in stage 5, but not significant. Compared with stage 4, Isoform0019016 was significantly downregulated in stage 5 (Figure 10D). However, the correlation heatmap displayed no obvious correlation between the expression levels of these two genes and flavonols contents (Figure 10E), so whether flavonols directly affect the expression of ABCB encoding genes needs further experiments to be proved. There were also data supporting the interaction between PIN1 and ABCB19 in *Arabidopsis*, but PIN1 and ABCB19 were not associated at any time in terms of the ratio of the two proteins in PIN1-GFP affinity precipitates (Teale et al., 2021). Meanwhile, biochemical data indicated that ABCBs and TWD1 were targets of the flavonol inhibition of polar auxin transport, while genetic data pointed to PIN1 (Mohanta et al., 2018), indicating that the process of flavonols regulating polar auxin transport was very complex. Moreover, the effects of flavonols on polar auxin transport and PIN localization varied with tissue and cell type (Kuhn et al., 2017).

At present, although no studies have reported the effect of flavonols on influx carrier AUX/LAX, six genes encoding AUX and six genes encoding LAX were found based on the results this study. AUX encoding gene expression changed irregularly in different stages (Figure 10A). However, most of the genes encoding LAX (Isoform0008732, Isoform0010377, Isoform0018938, and Isoform0002221) were downregulated in stage 4 but were upregulated in stage 5 (Figure 10B). We speculated that flavonols may bind to LAX, and that to maintain normal auxin transport, the system may promote the expression of genes encoding LAX, leading to the increase in its expression in stage 5. The correlation heatmap also showed a significant positive correlation between kaempferol content and the expression levels of Isoform0008732, Isoform0010377, Isoform0018938, and Isoform0002221, but this process still needs further studies. Interestingly, there was no significant correlation between myricetin content and these four genes expression levels. Previous studies have reported that the impact of different flavonols on PIN binding affinity and induction of PIN subcellular localization is different; for example, compared with other flavonols, morin can relatively effectively stabilize the PIN complex (Teale et al., 2021). Based on the results of this study, it can also be speculated that compared with myricetin, kaempferol has more effective impact on LAX gene expression, and that the regulatory mechanism of this process can also become the direction of further research in the future. Meanwhile, these results indicated that a large amount of flavonols was accumulated in stage 5 of *M. pasquieri*, and that flavonols may affect the expression of polar auxin transport carriers encoding genes at the transcriptional level, which may lead to the inhibition of polar auxin transport, resulting in slow growth.

Although the effects of flavonols on auxin transport have been studied from the perspective of proteomics and metabolomics (Geisler et al., 2016; Kuhn et al., 2017), so far, few studies have combined transcriptome and metabolome to study how flavonols affect polar auxin transport in post-germination growth. This is the novelty of this study, but it does not involve proteome analysis, which leads to certain limitations of the analysis. To further explore the mechanisms by which flavonols affect polar auxin transport, genetic approaches should be used in combination with computational analyses of large datasets, such as genomes, transcriptome, proteome, and metabolome, which may help identify new regulatory and related candidate relationships. In addition, the results of this study showed that 349 genes were found involved in the plant hormone signal transduction pathway, but that only one metabolite, salicylic acid, was found in the whole pathway, and that no auxin was detected (Supplementary Figure 7). This may be because of the relatively low content of plant hormones, and we performed widely targeted metabolomics, so they were undetected. Therefore, in the latter study, targeted metabolome detection methods should be used to detect plant hormone substances to better study the polar auxin transport and further verify the specific reasons for the slow growth of *M. pasquieri* seedlings. Furthermore, the samples were mixed samples of the whole organism, and there was no separate sampling and sequencing for different tissues, so the expression of transcription and metabolism of each tissue could not be determined in the later analysis, which hindered the study on the effect of flavonols on polar auxin transport in different tissues. In addition, the relationship between the binding affinity of different flavonols with different auxin transport carriers, the induction of their subcellular localization, and the inhibition intensity of polar auxin transport also needs to be further studied.

In summary, this study investigated *M. pasquieri* post-germination biological processes using a combination of transcriptomic and metabolomic methods. Using the morphological differential strategy, we have addressed the combination of molecular level and morphology better. The results of WGCNA showed that the flavonoid biosynthesis pathway was significantly enriched in stage 4, and that most of the key genes within the modules were also involved in this pathway. In addition, the transcriptome and metabolome association analysis showed that the genes encoding flavonol biosynthesis were significantly upregulated in stage 4 and promoted the accumulation of flavonols in stage 5, suggesting that the changes in metabolites were driven by the level of transcript. By analyzing the expression level of the genes encoding auxin transport carriers and its association with flavonol content, it was speculated that flavonols may directly inhibit the expression of the PIN4 encoding gene and indirectly affect other genes encoding polar auxin transport carriers in *M. pasquieri*, which might also explain the slow growth of the *M. pasquieri* seedlings. This study was the first to reveal the dynamic changes in DEGs and DAMs of *M. pasquieri* in the post-germination stages using the multi-omics method, which laid a foundation for the study on the growth and development of the seedlings of *M. pasquieri* at the molecular level and provided new insights for the protection of this rare and endangered plant.

## Data Availability Statement

The datasets presented in this study can be found in online repositories. The names of the repository/repositories and accession number(s) can be found at: https://www.ncbi.nlm.nih.gov/, BioProject ID: PRJNA639907.

## Author Contributions

LK and LZ designed the study. LK, QL, ZC, SW, and YM performed the experiments. LK analyzed and visualized the transcriptomic, metabolomic, and RT-qPCR data and drafted the manuscript. ZS and LZ revised the manuscript. All authors contributed to the article and approved the submitted version.

## Funding

This study was supported by Guangdong Wildlife Conservation and Management Projects (Grant Nos: YSDZW202001 and YSDZW2021) and Forestry Department of Guangdong Province, China, for noncommercial ecological forest research (Grant No: 2020STGYL001).

## Conflict of Interest

The authors declare that the research was conducted in the absence of any commercial or financial relationships that could be construed as a potential conflict of interest.

## Publisher's Note

All claims expressed in this article are solely those of the authors and do not necessarily represent those of their affiliated organizations, or those of the publisher, the editors and the reviewers. Any product that may be evaluated in this article, or claim that may be made by its manufacturer, is not guaranteed or endorsed by the publisher.
