# Supplementary material for: Dynamic Transcriptomic and Metabolomic Analyses of Madhuca pasquieri (Dubard) H. J. Lam During the Post-germination Stages
Source: Front Plant Sci. 2021 Sep 30;12:731203. doi: 10.3389/fpls.2021.731203 (PMC8516028; doi:10.3389/fpls.2021.731203)
Supplement: Supplementary file 1 [file Data_Sheet_1.ZIP › Supplementary files/Figure captions in Supplementary files.docx]

**Supplementary Figure 1 |** Overlapping analysis of the total ion current (TIC) in different quality control (QC) samples by positive **(A)** and negative **(B)** ions electrospray ionization. The abscissa represents the retention time (min) of metabolite detection, and the ordinate represents the intensity of the ion current (cps: count per second).

**Supplementary Figure 2 |** Plots from orthogonal projection to latent structures-discriminant analysis (OPLS-DA) for modeling the differences between stage 1 and stage 2 **(A)**, stage 1 and stage 3 **(B)**, stage 1 and stage 4 **(C)**, stage 1 and stage 5 **(D)**, stage 2 and stage 3 **(E)**, stage 2 and stage 4 **(F)**, stage 2 and stage 5 **(G)**, stage 3 and stage 4 **(H)**, stage 3 and stage 5 **(I)**, and stage 4 and stage5 **(J)** in *M. pasquieri*.

**Supplementary Figure 3 |** Heatmap of differentially accumulated metabolites (DAMs) between stage 1 and other groups (stage 2–5) in *M. pasquieri*. **(A)** Heatmap representing the hierarchical cluster analysis in stage 1 vs. stage 2. **(B)** Heatmap representing the hierarchical cluster analysis in stage 1 vs. stage 3. **(C)** Heatmap representing the hierarchical cluster analysis in stage 1 vs. stage 4. **(D)** Heatmap representing the hierarchical cluster analysis in stage 1 vs. stage 5. The colors from magenta to cyan indicates low to high accumulation.

**Supplementary Figure 4 |** KEGG pathway enrichment analysis of DAMs. **(A)** Pathway enrichment in stage 1 vs. stage 2. **(B)** Pathway enrichment in stage 1 vs. stage 3. **(C)** Pathway enrichment in stage 1 vs. stage 4. **(D)** Pathway enrichment in stage 1 vs. stage 5. The x-axis represents the enrichment factor, while the y-axis represents the enrichment pathway. The dot sizes represent the number of DAMs. The statistical analysis of the pathway enrichment was performed using Fisher’s exact test. A lower q-value indicates that a lower percentage of significant results will be false positives. Rich factor represents the degree of enrichment of genes under the designated pathway term. Greater the value of the rich factor, greater is the degree of pathway enrichment.

**Supplementary Figure 5 |** Principal component analysis (PCA) of the transcriptomic data in the five development stage samples; the x-axis represents the first principal component and the y-axis represents the second principal component.

**Supplementary Figure 6 |** KEGG pathway enrichment analysis of differentially expressed genes (DEGs). **(A)** Pathway enrichment in stage 1 vs. stage 2. **(B)** Pathway enrichment in stage 1 vs. stage 3. **(C)** Pathway enrichment in stage 1 vs. stage 4. **(D)** Pathway enrichment in stage 1 vs. stage 5. The x-axis represents the enrichment factor, while the y-axis represents the enrichment pathway. The dot sizes represent the number of DEGs. The statistical analysis of the pathway enrichment was performed using Fisher’s exact test. A lower q-value indicates that a lower percentage of significant results will be false positives. Rich factor represents the degree of enrichment of genes under the designated pathway term. Greater the value of the rich factor, greater is the degree of pathway enrichment.

**Supplementary Figure 7 |** Transcript and metabolic profiling of genes in plant hormone signal transduction pathway in *M. pasquieri*.
