# Supplementary figures and images for: Dynamic Transcriptomic and Metabolomic Analyses of Madhuca pasquieri (Dubard) H. J. Lam During the Post-germination Stages
Source: Front Plant Sci. 2021 Sep 30;12:731203. doi: 10.3389/fpls.2021.731203 (PMC8516028; doi:10.3389/fpls.2021.731203)

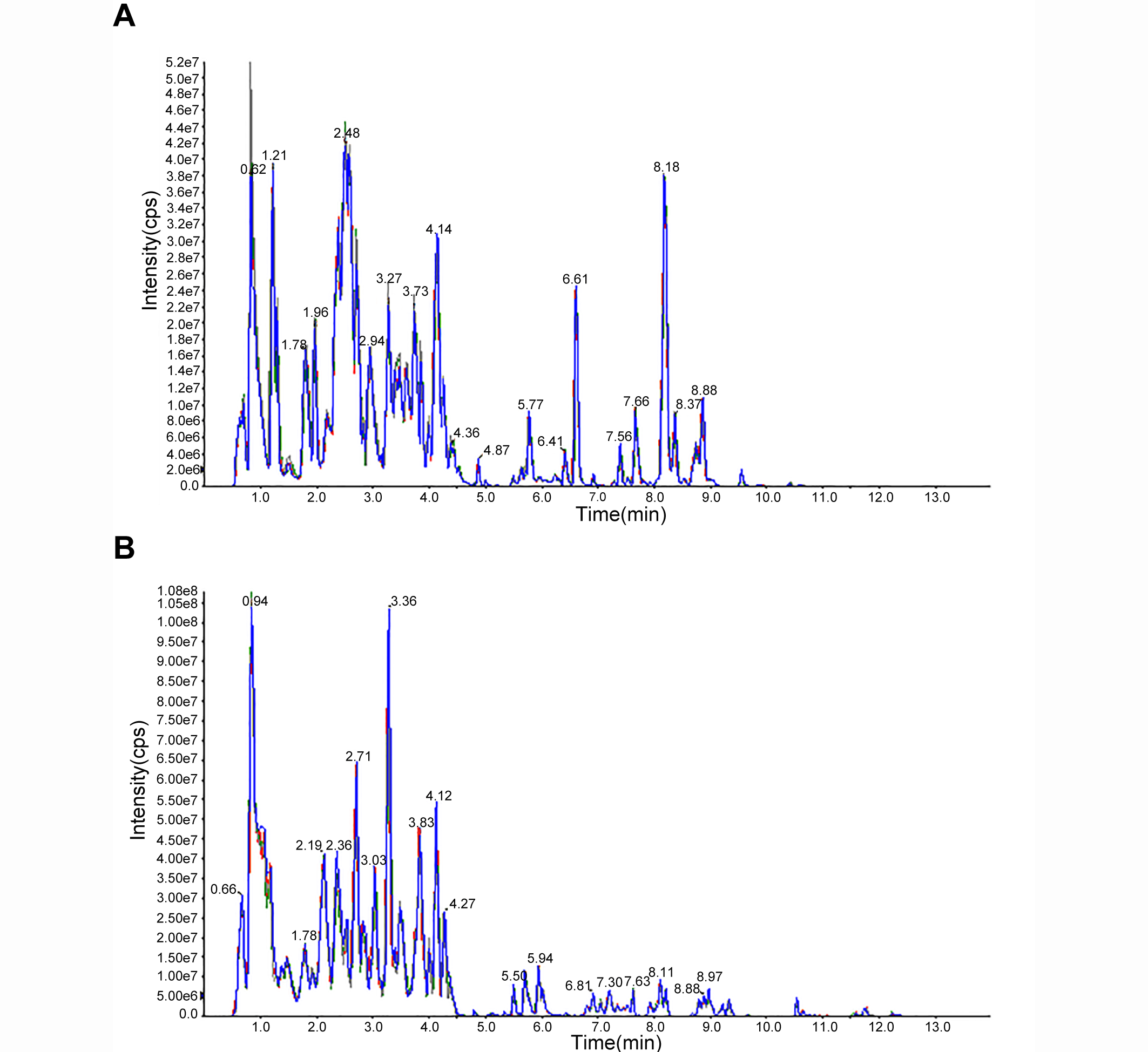

Supplement: Supplementary file 1 [file Data_Sheet_1.ZIP › Supplementary files/Figure S1.tif]

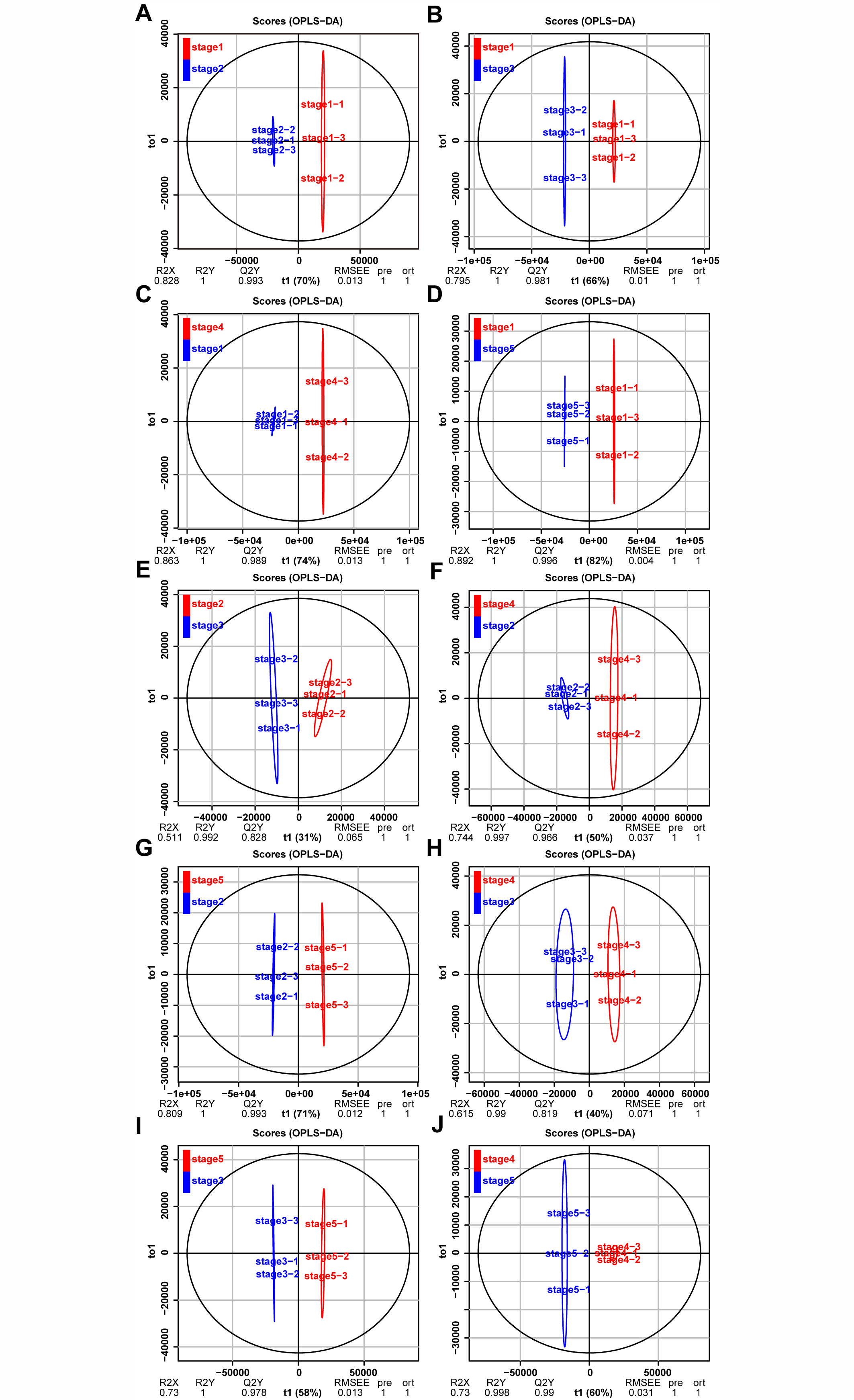

Supplement: Supplementary file 1 [file Data_Sheet_1.ZIP › Supplementary files/Figure S2.tif]

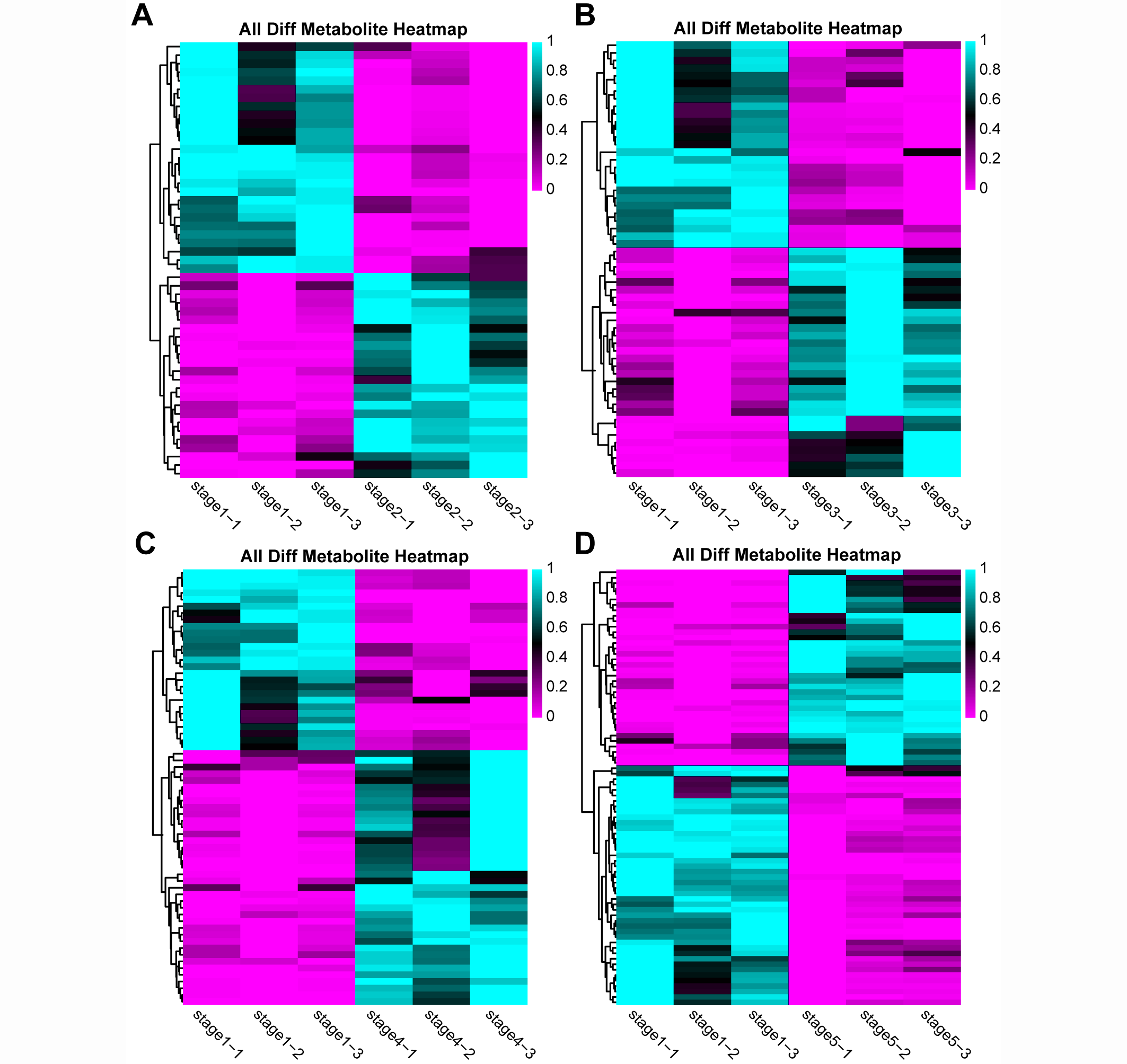

Supplement: Supplementary file 1 [file Data_Sheet_1.ZIP › Supplementary files/Figure S3.tif]

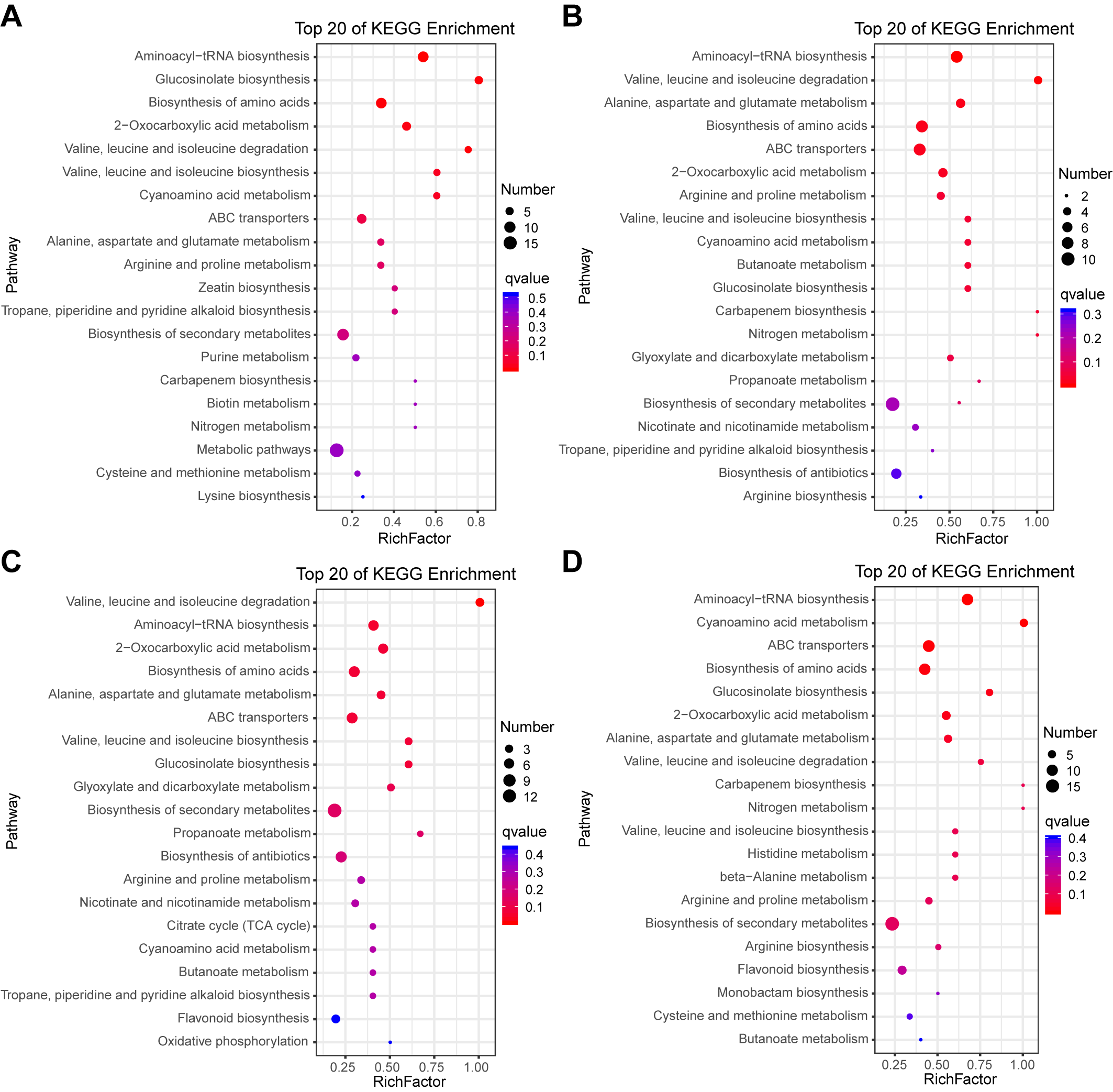

Supplement: Supplementary file 1 [file Data_Sheet_1.ZIP › Supplementary files/Figure S4.tif]

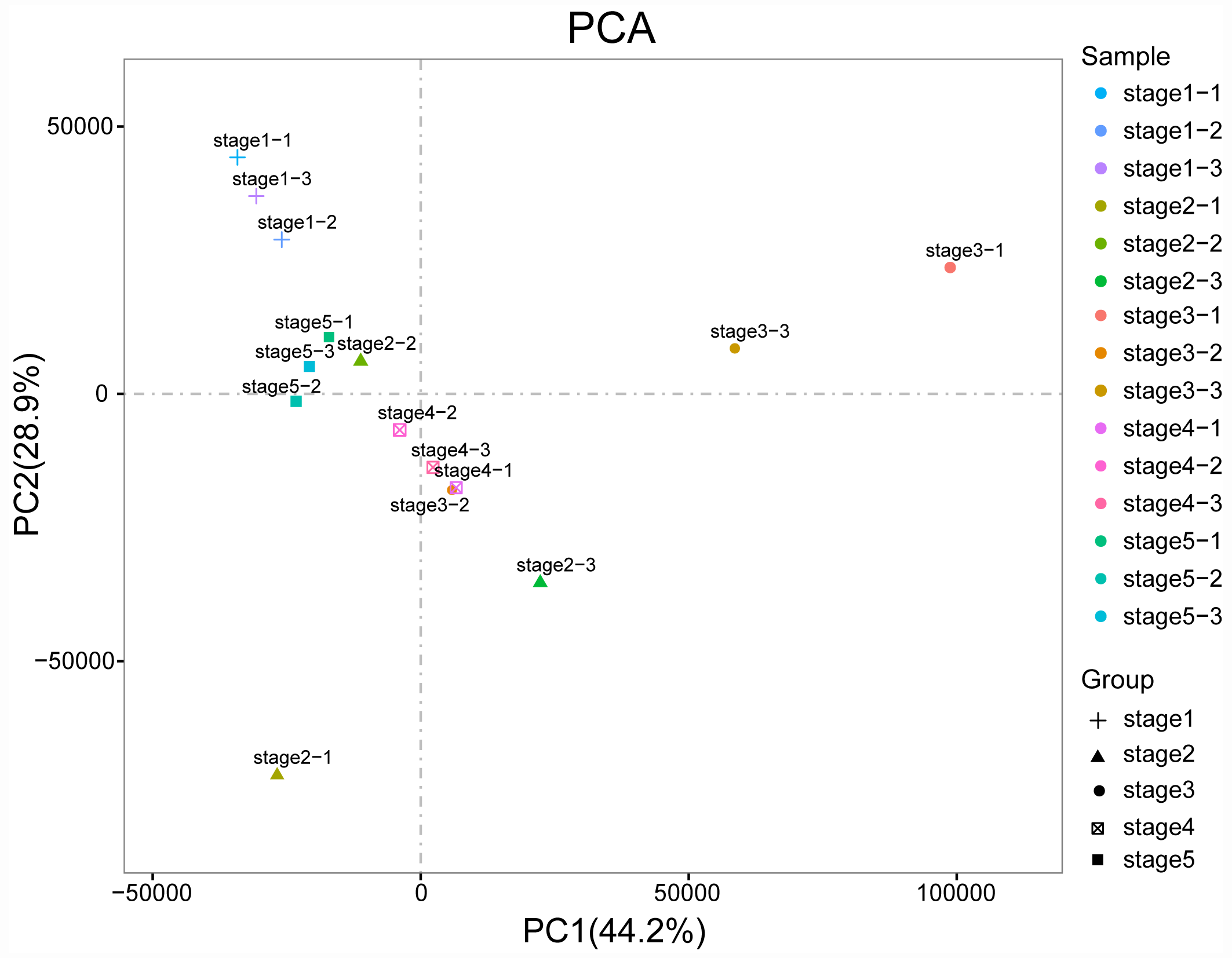

Supplement: Supplementary file 1 [file Data_Sheet_1.ZIP › Supplementary files/Figure S5.tif]

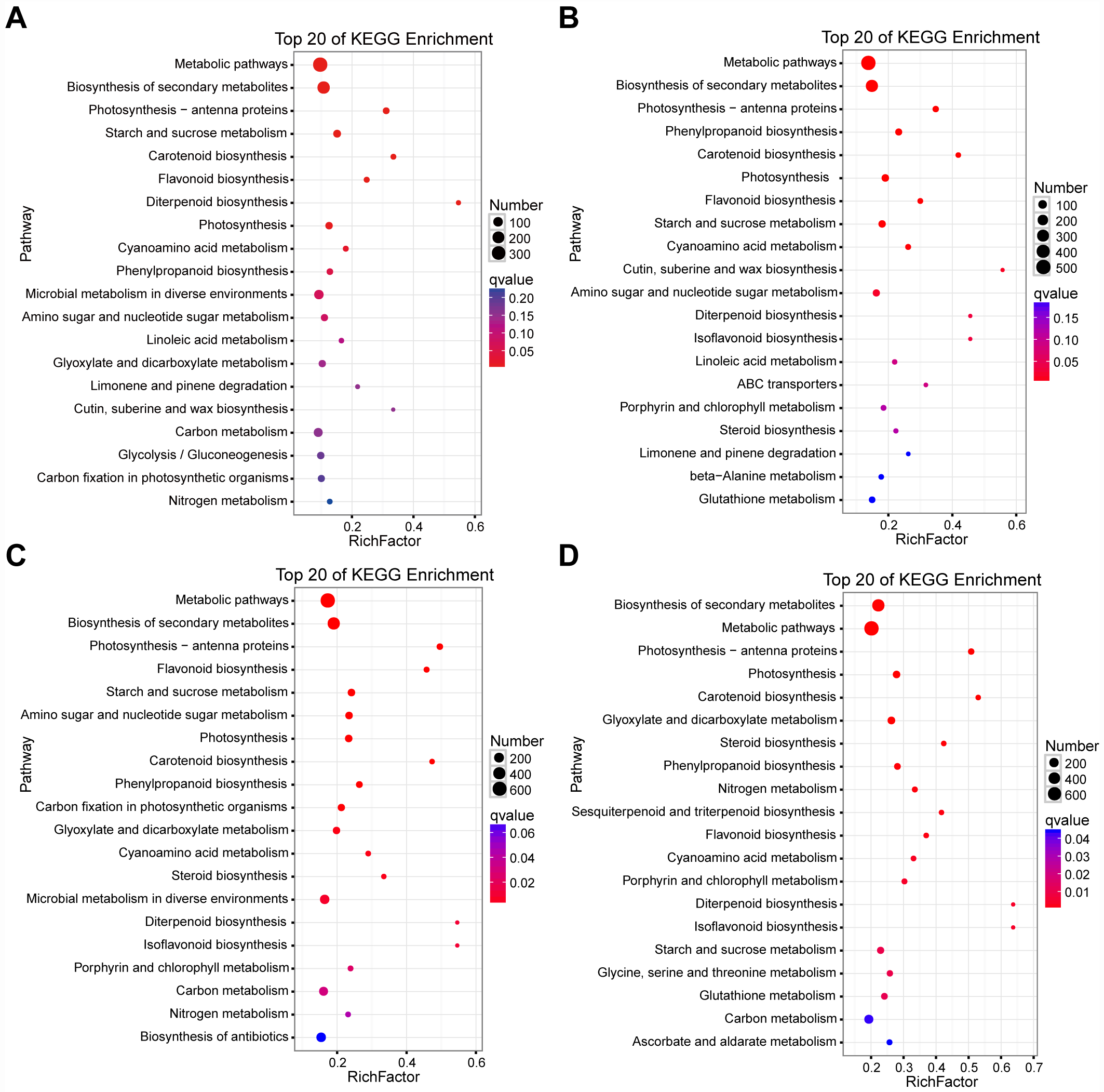

Supplement: Supplementary file 1 [file Data_Sheet_1.ZIP › Supplementary files/Figure S6.tif]

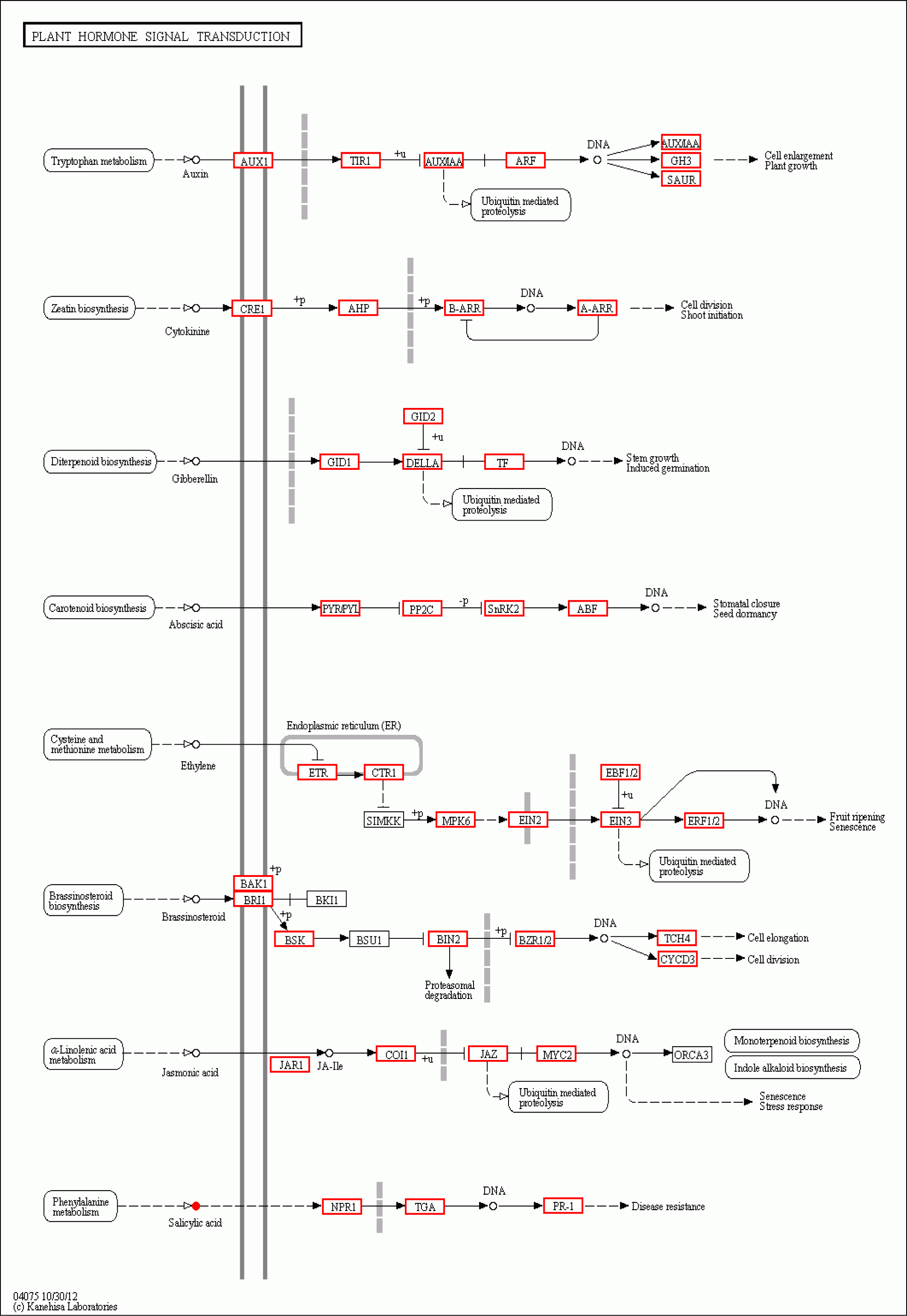

Supplement: Supplementary file 1 [file Data_Sheet_1.ZIP › Supplementary files/Figure S7.tif]
